# Analysis of Ribosome-Associated mRNAs in Rice Reveals the Importance of Transcript Size and GC Content in Translation

**DOI:** 10.1534/g3.116.036020

**Published:** 2016-11-14

**Authors:** Dongyan Zhao, John P. Hamilton, Michael Hardigan, Dongmei Yin, Tao He, Brieanne Vaillancourt, Mauricio Reynoso, Germain Pauluzzi, Scott Funkhouser, Yuehua Cui, Julia Bailey-Serres, Jiming Jiang, C. Robin Buell, Ning Jiang

**Affiliations:** * Department of Horticulture, Michigan State University, East Lansing, Michigan 48824; † Department of Plant Biology, Michigan State University, East Lansing, Michigan 48824; ‡ Department of Statistics and Probability, Michigan State University, East Lansing, Michigan 48824; § Department of Mathematics, San Francisco State University, California 94132; ** Center for Plant Cell Biology, University of California, Riverside, California 92521; †† Department of Botany and Plant Sciences, University of California, Riverside, California 92521; ‡‡ Genetics Program, Michigan State University, East Lansing, Michigan 48824; §§ Department of Horticulture, University of Wisconsin–Madison, Wisconsin 53705; *** Program in Ecology, Evolutionary Biology and Behavior, Michigan State University, East Lansing, Michigan 48824

**Keywords:** translating ribosome affinity purification sequencing, GC content, mRNA length, *Oryza sativa*

## Abstract

Gene expression is controlled at transcriptional and post-transcriptional levels including decoding of messenger RNA (mRNA) into polypeptides via ribosome-mediated translation. Translational regulation has been intensively studied in the model dicot plant *Arabidopsis thaliana*, and in this study, we assessed the translational status [proportion of steady-state mRNA associated with ribosomes] of mRNAs by Translating Ribosome Affinity Purification followed by mRNA-sequencing (TRAP-seq) in rice (*Oryza sativa*), a model monocot plant and the most important food crop. A survey of three tissues found that most transcribed rice genes are translated whereas few transposable elements are associated with ribosomes. Genes with short and GC-rich coding regions are overrepresented in ribosome-associated mRNAs, suggesting that the GC-richness characteristic of coding sequences in grasses may be an adaptation that favors efficient translation. Transcripts with retained introns and extended 5′ untranslated regions are underrepresented on ribosomes, and rice genes belonging to different evolutionary lineages exhibited differential enrichment on the ribosomes that was associated with GC content. Genes involved in photosynthesis and stress responses are preferentially associated with ribosomes, whereas genes in epigenetic regulation pathways are the least enriched on ribosomes. Such variation is more dramatic in rice than that in *Arabidopsis* and is correlated with the wide variation of GC content of transcripts in rice. Taken together, variation in the translation status of individual transcripts reflects important mechanisms of gene regulation, which may have a role in evolution and diversification.

Within a genome, a gene can encode a mRNA that encodes a protein or a noncoding RNA. Protein coding genes are first transcribed in the nucleus into a precursor mRNA (pre-mRNA) from which introns are cotranscriptionally or post-transcriptionally spliced, the 5′ end capped, and the 3′ end cleaved and polyadenylated. The processing of pre-mRNA leads to the generation of mature mRNAs that are transported to the cytoplasm and translated into proteins. Translation occurs on the ribosome complex and amino acids are provided to the nascent peptide chain via transfer RNAs (tRNAs). Not all mRNAs are translated at the same efficiency and some may be stored or degraded (Picard *et al.* 2012; Shah *et al.* 2013). The monitoring of not only the steady-state abundance of specific transcripts but also the level at which they are associated with ribosomes can provide insight into the regulation of gene expression. With advances in sequencing technology, sequencing of cDNAs that are derived from mRNAs (mRNA-seq) has become an efficient and convenient method to quantify transcript abundance, generally interpreted as gene expression levels. While analysis of mRNA-seq results provides a comprehensive atlas of the transcriptome, post-transcriptional regulation can affect the correlation between transcript and protein abundance. To more closely estimate protein levels, the abundance of individual mRNAs associated with the ribosome, referred to as the translatome (Halbeisen *et al.* 2009; Mustroph *et al.* 2009b), can be used as a proxy of the proteome. Actively translated mRNAs are associated with multiple ribosomes in polyribosome (polysome) complexes. As translation is largely regulated during the engagement of ribosomal subunits on the mRNA (initiation phase of translation) in plants and other organisms (Browning and Bailey-Serres 2015), an mRNA associated with polysomes is most likely to be undergoing the elongation and termination phases of translation. The mapping of the nuclease-protected footprints of individual ribosomes on mRNAs largely supports this conclusion, except for mRNAs with short upstream open reading frames (uORFs) that generally dampen translation of the main protein coding region of mRNAs (Juntawong *et al.* 2014). Intact ribosomes can be immunoprecipitated using an epitope-tagged large ribosomal protein subunit from which polysome-associated mRNAs are prepared, greatly reducing contamination of mRNAs associated with messenger ribonucleoproteins of density similar to ribosome complexes (Zanetti *et al.* 2005). When this approach is combined with mRNA-seq, the translation status of genes can be determined in a genome-wide fashion, a technique called TRAP-seq (Reynoso *et al.* 2015).

Rice is the world’s most important food crop, as it serves as a staple for more than half of the world’s population (Khush 2013). Among the cereal crops, rice has the smallest genome (389 Mb) (International Rice Genome Sequencing Project 2005), and is a model species for other cereal crops such as maize, sorghum, wheat, and barley. Rice, along with other cereals and in contrast to dicot plant species such as *Arabidopsis thaliana*, has a distinct bimodal distribution of genes with respect to GC content. For example, gene GC content in *Arabidopsis* ranges from 40–55%, with a nearly normal distribution (Wang and Hickey 2007). However, for the grasses (Poaceae) including rice, the genic GC distribution is more widely dispersed, ranging from 40 to 75% (Carels and Bernardi 2000; Wong *et al.* 2002). It has been proposed that GC-biased gene conversion (gBGC) is mainly responsible for the evolution of GC content of genes in monocots (Clement *et al.* 2015; Glemin *et al.* 2014). Previous studies have shown that GC-rich genes in rice are associated with more variable expression at the transcription level (Tatarinova *et al.* 2010) and GC-rich genes demonstrate stronger codon usage bias (Guo *et al.* 2007); nevertheless, the biological significance of varied GC content of rice (grass) genes is largely unclear, including its potential impact on translation.

The availability of translatome data for rice would greatly facilitate fundamental and applied research for both protein-coding genes and transposable elements, which are DNA sequence units that can rapidly change their locations and copy numbers in the genome. Previous proteomics studies on rice provided evidence of translation for a few thousand gene transcripts (Koller *et al.* 2002; Komatsu 2005). In addition, Park *et al.* (2012) conducted a genome-wide analysis of total and polysomal mRNAs using a rice 3′ tiling microarray. However, the focus of the study was on the influence of stresses on a subset of genes, and the translation status of the majority of individual rice genes is still unknown. In this study, we constructed an epitope-tagged rice Ribosomal Protein L18 cDNA line (*p35S:HF-OsRPL18*) to facilitate the isolation of polysomes and conduct TRAP-seq analysis. Findings made in this study provide new perspectives regarding the regulation of translation as well as guidance for future crop engineering.

## Materials and Methods

### Construction of the p35S:HF-OsRPL18 transgene and rice transformation

All genes described in this study are referred to by their MSU Rice Genome Annotation Project Release 7 gene identifier (http://rice.plantbiology.msu.edu/) (Kawahara *et al.* 2013). For construction of the *p35S:HF-OsRPL18* transgene, the coding region of LOC_Os03g22180 was amplified from rice cDNA, and the PCR product was cloned into the pENTR/D-TOPO TA vector (Invitrogen, Carlsbad, CA) and then recombined into the destination vector p35S:HF-GATA, which contains a His_6_-FLAG tag (Mustroph *et al.* 2010). Thereafter, the *HF-OsRPL18* sequence was amplified by PCR, recloned into the TA vector, and the *HF-OsRPL18* sequence was excised using *Sal*I and *Pst*I and then placed between the same restriction sites in pCAMBIA1300S, which contains a double 35S promoter upstream of the multiple cloning site (Xiong and Yang 2003). This yielded the expression construct containing *p35S:HF-OsRPL18* used for transformation of the rice cultivar Nipponbare.

The basal medium used for transformation was Murashige and Skoog (MS) (Murashige and Skoog 1962) supplemented with 30 g/L sucrose and Gelzan 3 g/L (PhytoTechnology Laboratories, Overland Park, KS) at pH 5.7. Callus cell growth was induced on callus induction medium (CIM, MS plus 2 mg/L 2,4-dichlorophenoxyacetic acid) using mature seeds and then cocultured for 2 d in the dark at 25° with *Agrobacterium tumefaciens* EHA 105 carrying the expression vector with *p35S:HF-OsRPL18* and 200 µM acetosyringone to enable infection. Each callus was transferred to selection medium (CIM plus 250 mg/L cefatoxime and 50 mg/L hygromycin B) to cease growth of *Ag. tumefaciens* and allow the growth of transgenic calli. Four to eight weeks after selection, each callus was transferred to selection regeneration medium (MS plus 250 mg/L cefatoxime, 50 mg/L hygromycin B, 3 mg/L 6-benzylaminopurine, and 0.5 mg/L 1-naphthaleneacetic acid) for seedling generation.

Plants generated from hygromycin-resistant calli were transferred to soil and allowed to self-pollinate. Two-wk-old T_1_ seedlings were tested for hygromycin resistance by placing 1 cm leaf tips on Whatman paper moistened with 50 mg/L hygromycin for 4 d. Tips from plants with successful transgene integration remained green (resistant) whereas those without the transgene turned brown (nonresistant). To determine the copy number of the T-DNA in transgenic plants, DNA was extracted from leaves of resistant T_1_ seedlings using the CTAB method (Doyle and Doyle 1987), and digested with *Sac*I to conduct Southern DNA blot analysis. The digested DNA was resolved on a 1% (w/v) agarose gel, followed by transfer of the DNA to a nylon membrane (GE Healthcare, Pittsburgh, PA) using capillary flow. A 419 bp fragment from the hygromycin resistance gene was amplified using a pair of primers (forward 5′-CAAGGAATCGGTCAATACACTAC-3′ and reverse 5′-AAGCTCTGATAGAGTTGGTCAAG-3′) designed from the expression vector and labeled with digoxigenin, which was detected by anti-digoxigenin-AP, Fab fragments (Roche Applied Science, Indianapolis, IN). The hygromycin-resistant T_1_ plants were advanced to the T_2_ generation and hygromycin resistance was tested with T_2_ seedlings. If all T_2_ plants from a single T_1_ plant were resistant and the relevant T_1_ plant only harbored a single integration event, the relevant line was considered homozygous. One such homozygous line was chosen for subsequent analysis (Supplemental Material, Figure S1).

### Plant material and growth

To examine the assembly of HF-OsRPL18 into polysomes, T_4_ transgenic rice seeds containing *p35S:HF-OsRPL18* were sterilized and grown on MS medium with 10 g/L sucrose. Plants were grown for 7 d at 28° with a 16 hr day/8 hr night photoperiod under 80 µmol/m^2^/sec photosynthetically active radiation.

For mRNA-seq and TRAP-seq analysis, 5-wk-old calli were harvested from T_4_ seeds grown on CIM. For shoot and panicle mRNA-seq and TRAP-seq, T_4_ *p35S:HF-OsRPL18* plants were grown in 5 inch plastic pots filled with Profile Green’s Grade sand. The pots were placed inside a tray filled with water. Fertilizer (Peters Excel 15-5-15 Cal-Mag special) was applied twice each week as a 200 ml solution from a 10× stock to each tray with eight pots. All plants were grown in a growth chamber with a 12 hr day/12 hr night photoperiod, with light intensity 500 µmol/m^2^/sec; the above-ground portion of young seedlings (shoots) was harvested at 14 d from plants grown at 30/28° d/night; young panicles of 1–5 cm in length, corresponding to stage R1 (post the formation of panicle branches) according to Counce *et al.* (2000), were harvested from plants grown at 28/24° d/night. All tissues were harvested directly into liquid nitrogen and stored at −80° until use.

### Sucrose density fractionation of polysomes and efficiency evaluation of ribosome immunopurification

To test the association of HF-OsRPL18 with translating ribosomes, polysomal complexes were isolated by differential centrifugation and processed as described previously (Mustroph *et al.* 2009a). Approximately 1 g of frozen, pulverized 7-d-old shoot tissue was used to obtain a pellet enriched in ribosomes. About 3300 OD_260_ were fractionated on a 20–60% (w/v) sucrose density gradient. Protein in each fraction was precipitated and polypeptides analyzed by standard western blot after separation by 12% (w/v) SDS-PAGE. HF-OsRPL18 was immunodetected using a mouse monoclonal antibody conjugated to horseradish peroxidase (α-FLAG-HRP; 1:1000) as previously described (Zanetti *et al.* 2005). The small ribosomal subunit protein RPS6 was immunodetected using a rabbit polyclonal antiserum against *Zea mays* RPS6 (1:5000) (Williams *et al.* 2003) as the primary antibody and a horseradish peroxidase conjugated goat anti-rabbit IgG as the secondary antibody (1:10,000; Bio-Rad, Hercules CA).

To test the efficiency of immunopurification, ∼30 mg of pulverized shoot tissue of wild-type (Nipponbare) or *p35S:HF-OsRPL18* plants was processed for affinity purification of epitope-tagged ribosomes as described by Mustroph *et al.* (2009a) with the following modification: 5 µg of α-FLAG (Sigma-Aldrich, St. Louis MO, F1804) was bound to 50 µl Dynabeads Protein G (Life Technologies, Carlsbad CA, 1004D) instead of α-FLAG agarose beads (Sigma-Aldrich, F2426). Fractions obtained during the procedure were separated by 12% (w/v) SDS-PAGE followed by western blot detection of HF-OsRPL18 and RPS6 as described above.

### mRNA-seq and TRAP-seq library construction, sequencing, and expression abundance estimation

For mRNA library construction, ∼5 g of pulverized tissue (shoot, callus, or young panicle) was homogenized in 45 ml of Polysome Extraction Buffer (PEB) and centrifuged at 4°, 16,000 × *g*, for 15 min (Mustroph *et al.* 2009a). The supernatant was filtered through Miracloth and centrifuged as described above. Five percent of the supernatant was used for isolation of total RNAs with the remainder used for affinity purification of epitope-tagged ribosomes as described previously (Mustroph *et al.* 2009a). RNAs were extracted from the PEB supernatant (for mRNA-seq) or the eluate from the immunoprecipitation (for TRAP-seq) using TRIzol reagent (Invitrogen) and the QIAGEN RNeasy kit.

Illumina TruSeq stranded mRNA libraries (Illumina, San Diego, CA) were constructed and sequenced on an Illumina HiSeq2500 (Illumina,) in paired-end mode for 100 or 150 nucleotides (nt) at the Research Technology Support Facility of Michigan State University. The read lengths and yield of each library are summarized in Table S1. Reads from libraries sequenced with read lengths greater than 100 nt were trimmed to 100 nt for consistency. The reads for each library were adapter trimmed and quality filtered using Trimmomatic (v0.32) in paired mode using the parameters: LEADING:5 TRAILING:5 SLIDINGWINDOW:4:10 MINLEN:30 (Bolger *et al.* 2014).

The cleaned paired reads from the mRNA-seq and TRAP-seq libraries were aligned to the rice pseudomolecules (Os-Nipponbare-Reference-IRGSP-1.0) (Kawahara *et al.* 2013) using TopHat (v1.4.1) (Trapnell *et al.* 2009) with a minimum intron size of 5 bp and a maximum intron size of 15 kbp. Transcript abundances for the MSU Release 7 representative gene models (Kawahara *et al.* 2013) were calculated as fragments per kilobase per exon model per million mapped reads (FPKMs) from the TopHat alignments using Cufflinks (v1.3.0) (Trapnell *et al.* 2010) with the same intron parameters as used for TopHat (Table S2).

### Translatomes, translatome enrichment index (TEI), and filtering of transposon genes

For each tissue, the translatome was comprised of all genes with a TRAP-seq FPKM value ≥1. The TEI was calculated as the ratio of the TRAP-seq FPKM *vs.* the mRNA-seq FPKM from the same tissue. TEIs of different gene groups (see below) were compared using the Kolmogorov–Smirnov (KS) test, and *P*-values were adjusted using the Benjamini–Hochberg method (Benjamini and Hochberg 1995). Representative gene models from MSU Release 7 (ftp://ftp.plantbiology.msu.edu/pub/data/Eukaryotic_Projects/o_sativa/annotation_dbs/pseudomolecules/version_7.0/all.dir/all.locus_brief_info.7.0) were used for all analyses, with the exception of analysis of alternative splice isoforms in which all gene models were considered. In MSU Release 7, 16,937 transposon genes (including both transposons and retrotransposons) were annotated. To confirm that transposon-related genes were correctly tagged, a custom rice transposon library was used to mask the predicted cDNAs of the 16,937 transposon genes. If 50% or more of the cDNA length was masked by the library, the transcript was validated as a true transposon gene; 15,461 transposon-related genes met this criterion and were used for subsequent analyses.

### Classification of genes by expression breadth and abundance

To determine the expression breadth of nontransposon genes, 23 mRNA-seq samples were downloaded from the National Center for Biotechnology Information (NCBI) Sequence Read Archive (SRA) (http://www.ncbi.nlm.nih.gov/sra). After exclusion of redundant samples (those from similar tissues), 12 distinct mRNA-seq samples were retained, in addition to the three mRNA-seq samples from this study, resulting in a total of 15 samples for the expression breadth analysis (Table S3). The SRA-obtained raw reads were cleaned, aligned to the reference genome as described above, and expression abundance was estimated as described above with an FPKM value ≥1 considered as expressed. Nontransposon genes were categorized into three groups with an increasing order of expression breadth: Breadth Group 1 comprised nontransposon genes expressed in 1–7 samples; Breadth Group 2 were nontransposon genes expressed in 8–14 samples; and Breadth Group 3 consisted of nontransposon genes with expression evidence in all 15 samples and were considered constitutively expressed genes.

The three mRNA-seq datasets generated in this study were used to classify nontransposon genes based on their expression abundance for each tissue. In each tissue, nontransposon genes were categorized into six groups with FPKM values increasing from 1 to over 1000. Each group contains comparable number of genes (Table S4).

### Identification of conserved and lineage-specific genes

Rice genes were assigned to a green plant lineage based on their placement within orthologous gene clusters generated with OrthoMCL (v1) (Li *et al.* 2003). OrthoMCL was used to cluster protein sequences from 13 species: *Amborella trichopoda* (v1.0, phytozome.jgi.doe.gov), *Aquilegia coerulea* (v1.1, phytozome.jgi.doe.gov), *A. thaliana* (TAIR10, phytozome.jgi.doe.gov), *Brachypodium distachyon* (v2.1, phytozome.jgi.doe.gov), *Musa acuminata* (v1, banana-genome.cirad.fr), *Oryza sativa* (v7, rice.plantbiology.msu.edu), *Phoenix dactylifera* (v3, Qatar-weill.cornell.edu/research/datepalmGenome), *Phyllostachys heterocycla* (v1.0, bamboogdb.org), *Populus trichocarpa* (v3.0, phytozome.jgi.doe.gov), *Setaria italica* (v2.1, phytozome.jgi.doe.gov), *Solanum lycopersicum* (iTAG2.3, phytozome.jgi.doe.gov), *Sorghum bicolor* (v2.1, phytozome.jgi.doe.gov), and *Z. mays* (5b; maizegdb.org). Based on species content, orthologous gene clusters were classified as conserved in all angiosperms, monocot and dicot-restricted, monocot-restricted, Poaceae-restricted, or *O. sativa*-specific. Transposon-related genes were not assigned a lineage. To visualize the relative translation status (or TEI) of expressed genes in each lineage, kernel density plots were generated for the three tissue types (shoot, callus, and panicle) surveyed. Nucleotide content for gene and coding sequences (CDS) was calculated using BEDTools (v2.20.1) (Quinlan and Hall 2010).

### Gene ontology (GO) analysis

GO assignment table of rice genes (Release 7) was downloaded from the Rice Genome Annotation Project (http://rice.plantbiology.msu.edu/), from which, genes were grouped according to their associated biological processes. The assignment table also contains the corresponding *Arabidopsis* orthologous gene for each rice gene, which was used to synchronize the analysis between rice and *Arabidopsis* (see below). Information of gene features, including length and GC content of CDS and untranslated regions (UTRs), were obtained based on the rice genome annotation (Release 7), and only genes with both 5′ UTRs (at least 20 bp in length) and 3′ UTRs (at least 110 bp in length) were retained for the following analysis. The minimum length of UTRs (20 bp for 5′ UTRs and 110 bp for 3′ UTRs) was defined using genes with full-length cDNA evidence (Kikuchi *et al.* 2003b), where 97.5% of these gene have UTRs equal or longer than the minimum length. These values were used for all analysis related to the specific roles of UTRs in translation. For each tissue, the mean values of TEIs of all genes within each category (each GO term) were calculated and only the categories with at least 40 genes were included in the comparison.

Similar analyses were conducted in *Arabidopsis* using mRNA-seq levels and TRAP-seq levels published from *Arabidopsis* seedlings under normal conditions (Juntawong *et al.* 2014). The CDS of *Arabidopsis* genes were downloaded from TAIR10 (https://www.arabidopsis.org/). To ensure comparability between *Arabidopsis* and rice, only genes corresponding to the genes used for GO analysis in rice shoots with TEIs were included (7966 genes for *Arabidopsis* and 11,600 for rice). Only processes with 40 or more genes were considered for comparison, as described above.

### Estimation of recombination rate and codon usage frequency

The recombination rate in rice was adapted from Tian *et al.* (2009), where 3982 markers were anchored to the rice genome, IRGSP Build 4.0 pseudomolecules. To be consistent with the other analyses conducted in this study, these markers were aligned to the rice genome MSU Release 7, where markers having the best match with ≥ 99% identity over the entire marker sequences were used for the following analysis. The recombination rate (cM/Mb) was measured in 1 Mb bins (some bins are >1 Mb if there is no marker in a region >1 Mb). Pearson correlation between recombination rate and CDS GC content, as well as that between recombination rate and TEI, were conducted using SAS/9.3.

The occurrence frequency of all 64 codons (61 for amino acids and 3 for stop codon) was determined using a perl script kindly provided by Kevin Childs and Tiffany Liu (Michigan State University). Briefly, codon usage frequency was calculated for genes with the lowest (<0.2) and the highest (≥3.0) TEIs and synonymous codon usage between the two gene groups were compared; chi-square tests were conducted using SAS/9.3.

### Data availability

RNA-sequencing reads (mRNA-seq and TRAP-seq) are available in the NCBI SRA under BioProject PRJNA298638. The mRNA-seq and TRAP-seq expression abundances are available in Table S2 and on individual gene report pages at the Michigan State University Rice Genome Annotation Project (http://rice.plantbiology.msu.edu/).

## Results and Discussion

### Expression of epitope-tagged RPL18 and its incorporation into ribosomes

In *A. thaliana*, it has been demonstrated that HF-AtRPL18 is efficiently incorporated into the 60S subunit and that ribosomes containing HF-AtRPL18 accumulate in polysomal complexes (Zanetti *et al.* 2005). To test whether this can be replicated in rice, we constructed an expression vector that contains the orthologous rice *RPL18* gene with a His_6_-FLAG tag at the N-terminus of the protein (HF-OsRPL18, see *Materials and Methods* for details). Transgenic *p35S:HF-OsRPL18* rice plants used for subsequent analyses contained a single copy of the transgene and were similar to the wild-type Nipponbare plants in terms of plant height, days to flower, number of tillers, seed number per plant, and seed weight (Figure S1).

**Figure 1:**
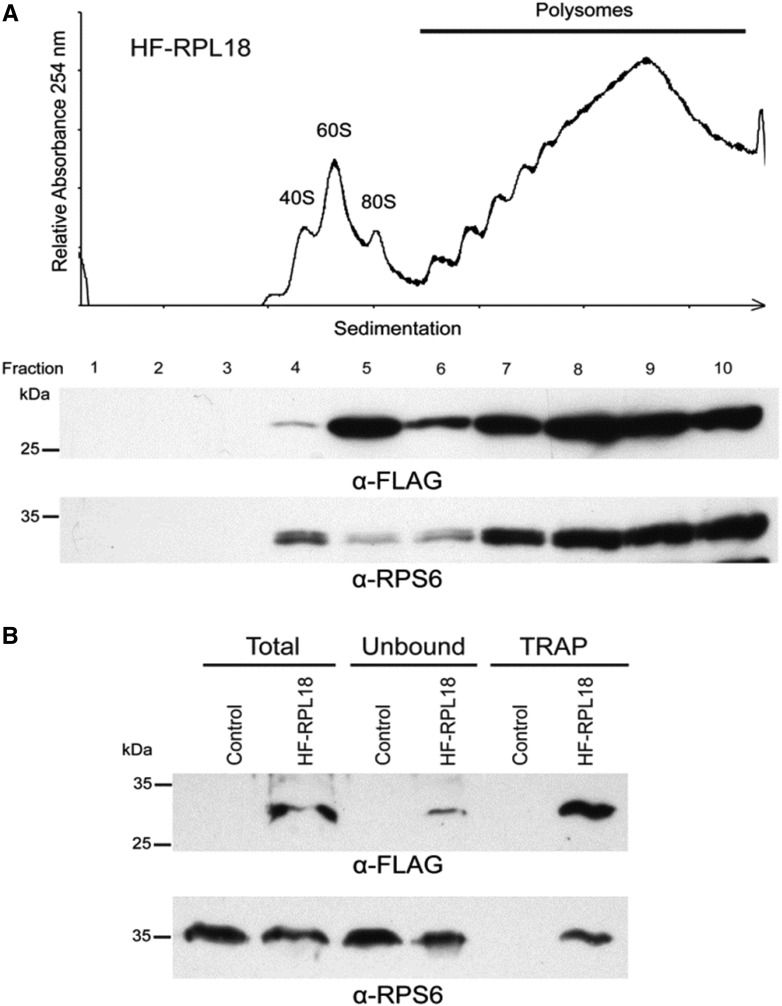
Epitope-tagged rice RPL18 assembles into functional ribosomes that can be purified by TRAP. (A) Confirmation of RPL18 assembly into ribosomes. The *p35S:HF-OsRPL18* rice line was used as the source of ribosomal complexes, which were separated by ultracentrifugation on a 20–60% (w/v) sucrose density gradient. The absorbance at 254 nm was recorded to detect the ribosomal subunits of 40S and 60S, monosomes (80S), and polysomes. The gradient was fractionated and proteins in the 10 fractions were analyzed by SDS-PAGE separation and western blotting processed with anti-FLAG (α-FLAG) or anti-RPS6 (α-RPS6) antisera. Molecular mass markers are indicated on the left. (B) Purification of polysomes by TRAP. Equal weights of pulverized tissue from untransformed Nipponbare (control) and homozygous transgenic *p35S:HF-OsRPL18* shoots were solubilized in polysome extraction buffer to obtain a clarified supernatant (total). The extract was incubated with anti-FLAG-bound Dynabeads coupled to Protein G to bind HF-RPL18. The supernatant (unbound fraction) was collected to evaluate the efficiency of the immunopurification. The magnetically captured protein–RNA complexes (TRAP fraction) was eluted from the beads using 3X-FLAG peptide. Each fraction was analyzed by western blot with α-FLAG and α-RPS6. The expected molecular mass of HF-RPL18 is 25 kDa. SDS-PAGE, sodium dodecyl sulfate-polyacrylamide gel electrophoresis; TRAP, translating ribosome affinity purification.

Two experiments were performed to confirm that the FLAG-epitope-tagged OsRPL18 was appropriately assembled into functional ribosomes that can be efficiently purified by TRAP. In the first experiment, the association of HF-OsRPL18 with the large ribosomal subunit and multi-ribosome complexes (polysomes) was confirmed by preparation of a shoot extract with a polysome-stabilizing buffer followed by pelleting of ribosome complexes at 170,000 × *g*, and further fractionation of the complexes by centrifugation through a sucrose density gradient. A UV absorbance profile of a representative gradient was obtained and western blot analysis was performed to evaluate the fractionation of HF-OsRPL18 and the core 40S ribosomal subunit protein RPS6. Both RPL18 and RPS6 had similar abundance in the small-to-large polysome complexes (Figure 1A; fractions 6–10), confirming assembly of the epitope-tagged protein into functional ribosomes. RPS6 and HF-OsRPL18 were enriched in fractions 4 and 5, respectively, consistent with their independent assembly into the 40S and 60S subunits, which separated into distinct peaks in the sucrose density gradient.

The second experiment was carried out to determine if ribosomes with HF-OsRPL18 could be directly and efficiently immunopurified from cell extracts prepared with polysome-stabilizing buffer. To accomplish this, the presence of HF-OsRPL18 and the 40S subunit RPS6 were monitored in the initial crude extract (total) and in the proteins obtained by immunopurification of FLAG-tagged HF-OsRPL18 and associated proteins using magnetic beads conjugated to an anti-FLAG monoclonal antibody (TRAP). As a control for the efficiency of the purification, the supernatant from the immunopurification (unbound) was also evaluated in the western blot analysis. Figure 1B shows a representative experiment that demonstrates that HF-OsRPL18 is efficiently purified along with RPS6, consistent with isolation of the 40S and 60S subunits in a complete 80S ribosome. A small amount of HF-OsRPL18 remained in the supernatant, even when an excess of antibody was used in the purification. This suggests that a small proportion of HF-OsRPL18-containing ribosomes may not have the epitope accessible to the immune-affinity matrix. RPS6 was also present in the unbound fraction. This was anticipated because 40S ribosomal subunits not coupled with HF-OsRPL18-containing 60S subunits would not be purified, as the *p35S:HF-OsRPL18* transgene was expressed in the wild-type Nipponbare background with three endogenous *RPL18* genes that are all expressed in shoots. These experiments confirm that TRAP can be used efficiently to purify functional ribosomes from *p35S:HF-OsRPL18* transgenic rice.

### Rice transcriptome and translatome profiles

To elucidate the regulation of rice gene expression at the transcriptional and translational levels, we conducted mRNA-seq and TRAP-seq with three different rice tissues (shoot, callus, and panicle) using the *p35SHF-OsRPL18* line. Rice genes were categorized into transposon and nontransposon gene sets to validate the efficiency of transcription (mRNA-seq) and translation (TRAP-seq). In this study, 16,937 transposon-encoding genes are annotated in Release 7 and we performed further filtering to yield a total of 15,461 transposon-related genes (see *Materials and Methods*), of which the majority encode a putative transposase or other proteins required for transposition and are therefore putative autonomous transposons or derivatives from autonomous elements. The nontransposon gene set contains 39,049 nontransposon protein-coding genes.

For simplicity, we defined a gene as transcribed (for mRNA-seq) and translated (for TRAP-seq) if it has an FPKM value ≥1. Moreover, we defined the ratio of FPKM values between TRAP-seq and mRNA-seq as a TEI of mRNAs associated with ribosomes (Hupe *et al.* 2014). As shown in Table 1, over half (55%) of the nontransposon genes have evidence of translation in at least one of the three tissues sampled. If only genes with transcription evidence (*i.e.*, mRNA-seq ≥ 1 FPKM) are considered, ∼90% of the transcribed nontransposon genes are associated with ribosomes, suggesting that, once transcribed, the majority of nontransposon genes are recruited to ribosomes for translation (Table 1). Of the 22,896 nontransposon genes with an annotated function in Release 7, 16,151 (71%) are translated in at least one of the three tissues examined, suggesting that most of them are true protein-coding genes consistent with their similarity to known genes. In the rice genome, 16,153 genes are annotated as “expressed proteins” or “hypothetical proteins”: of these, 5496 (34%) are translated in at least one of the three tissues examined. This suggests that, of the unknown proteins annotated solely based on transcription evidence and/or *ab initio* gene predictions, a substantial portion could be *bona fide* protein-coding genes. For a comparison, we examined 4095 genes with proteomic evidence in OryzaPG-DB (Helmy *et al.* 2012); 3884 (94.8%) have a TRAP-seq FPKM value ≥1 in this study. Nontransposon genes with transcripts that were not detected in this study may be functional in tissues not evaluated in this study.

**Table 1:** Gene expression evidence from transcriptomes and translatomes of three tissues of rice

	No. of Genes	Fraction of gene set	Callus	Panicle	Shoot	Total Unique Genes
Nontransposon genes	39,049	mRNA-seq (%total)	18,793 (48.13%)	19,311 (49.45%)	17,713 (45.36%)	22,589 (57.85%)
		TRAP-seq (%total)	18,013 (46.13%)	17,987 (46.06%)	15,943 (40.83%)	21,647 (55.44%)
		Overlap (%mRNA-seq^b^)	16,931 (90.09%)	16,804 (87.02%)	15,030 (84.85%)	20,457 (90.56%)
Transposon genes	15,461	mRNA-seq (%total)	293 (1.90%)	255 (1.65%)	202 (1.31%)	381 (2.46%)
		TRAP-seq (%total)	148 (0.96%)	149 (0.96%)	85 (0.55%)	211 (1.36%)
		Overlap (%mRNA-seq)	138 (47.10%)	122 (48.03%)	77 (38.31%)	180 (47.24%)

No., number; mRNA, messenger RNA; TRAP-seq, Translating Ribosome Affinity Purification followed by mRNA-sequencing; %total, the percent of annotated genes with mRNA-seq or TRAP-seq FPKM ≥ 1; %mRNA-seq, the percent that are translated among the genes with transcription evidence.

In contrast to nontransposon genes, very few transposon genes are transcribed and even fewer are translated (2.5 and 1.4% of the total transposon genes, respectively). Less than half (48%) of the transcribed transposons are translated compared to 90% for nontransposon genes (Table 1), suggesting that transcripts encoding transposon proteins are much less likely to be translated than nontransposon transcripts. Among the transposons that are translated, the median TEI value is 0.65, which is substantially lower than that of nontransposon genes (0.93). To assess whether the lower fraction of translated transposon genes is an artifact of low sensitivity of TRAP-seq, we examined the translation status of autonomous transposons reported to be active in Nipponbare. Among the nine transposons with reported activity in Nipponbare (Hirochika *et al.* 1996; Jiang *et al.* 2003; Kikuchi *et al.* 2003a; Nakazaki *et al.* 2003; Picault *et al.* 2009; Sabot *et al.* 2011), translational evidence was detected for six in this study (Table 2). The distinction in translatome profiles between transposons and nontransposon genes, high concordance of translatome evidence with genes supported by previous proteomic studies, and detection of the most active transposons in the translatome suggests that the TRAP-seq data generated in this study effectively separates mRNAs associated with ribosomes from pre/mRNAs in the nucleus or untranslated mRNAs in the cytosol.

**Table 2:** Translational status of transposable elements documented to be active in Nipponbare

Element	Superfamily	Locus	Tissue*^a^*	mRNA-seq (FPKM)	TRAP-seq (FPKM)	TEI	Translation Evidence*^b^*
*Tos17*	*LTR/Copia*	LOC_Os10g29650	Callus	15.03	0.58	0.04	Maybe
*Lullaby*	*LTR/Copia*	LOC_Os06g38480	Callus	13.01	15.61	1.20	Yes
*Osr10*	*LTR/Copia*	LOC_Os04g32750	Panicle	0.01	0.00	NA	Not expressed
*Osr37*	*LTR/Gypsy*	LOC_Os03g43750	Callus	10.12	4.83	0.48	Yes
*RIRE2*	*LTR/Gypsy*	LOC_Os03g36279	Callus	2.26	2.56	1.14	Yes
*RIRE3*	*LTR/Gypsy*	LOC_Os06g32890	Shoot	0.13	0.08	NA	Not expressed
*RN363*	*LTR/Gypsy*	LOC_Os02g24150	Panicle	1.92	2.02	1.05	Yes
*Ping*	*PIF/Harbinger*	LOC_Os06g39624	Panicle	16.50	9.41	0.57	Yes
*Pong*	*PIF/Harbinger*	LOC_Os11g19864	Callus	6.40	6.36	0.99	Yes

mRNA-seq, messenger RNA sequencing; FPKM, fragments per kilobase per exon model per million mapped reads; TRAP-seq, Translating Ribosome Affinity Purification followed by mRNA-sequencing; TEI, translatome enrichment index; NA, not applicable.

a If the transposon is expressed in multiple tissues, the one with the highest TRAP-seq level is listed.

b TRAP-seq FPKM ≥ 1 is considered to be translated; mRNA-seq FPKM ≥1 is considered to be expressed.

It should be noted that a high TEI could be interpreted in two ways as the abundance of mRNA on ribosomes is largely determined by three factors: initiation, elongation, and termination. The predominant regulation is the rate of initiation of translation, which is positively correlated with protein production (Browning and Bailey-Serres 2015). If the rate of elongation and termination is comparable between transcripts, then a high TEI indicates preferential translation, *i.e.*, translation is initialized on a higher fraction of available transcripts than for other individual mRNAs. Translation can be initialized by newly recruited ribosomes (*de novo* initiation) or, when translation is completed on an mRNA, the recently terminated ribosomes may reinitiate translation on the same mRNA, a process called ribosome recycling (Browning and Bailey-Serres 2015; Roy and von Arnim 2013; Shah *et al.* 2013). However, a high TEI could be due to other factors. One is a slower rate of translation elongation or termination, such that more transcripts are associated with ribosomes (Browning and Bailey-Serres 2015; Roy and von Arnim 2013). Factors including the structure of the mRNA coding region, abundance of tRNAs, and codon usage contribute to the variation of elongation speed (Ingolia 2014). Another factor is the presence of one or more upstream ORFs (uORFs) preceding the main protein CDS on the transcript. uORFs in the 5′ region of transcripts typically reduce the overall translation of the main CDS, in a metabolite or condition-regulated manner (Juntawong *et al.* 2014; Roy and von Arnim 2013). Moreover, these contributions to TEI are not mutually exclusive. Although slow elongation/termination rate or uORFs may contribute to high TEI values, thereby uncoupling this statistic from protein production, a very low TEI most likely indicates limited translation.

### Translatome enrichment associated with a short coding region and higher GC content

**Figure 2:**
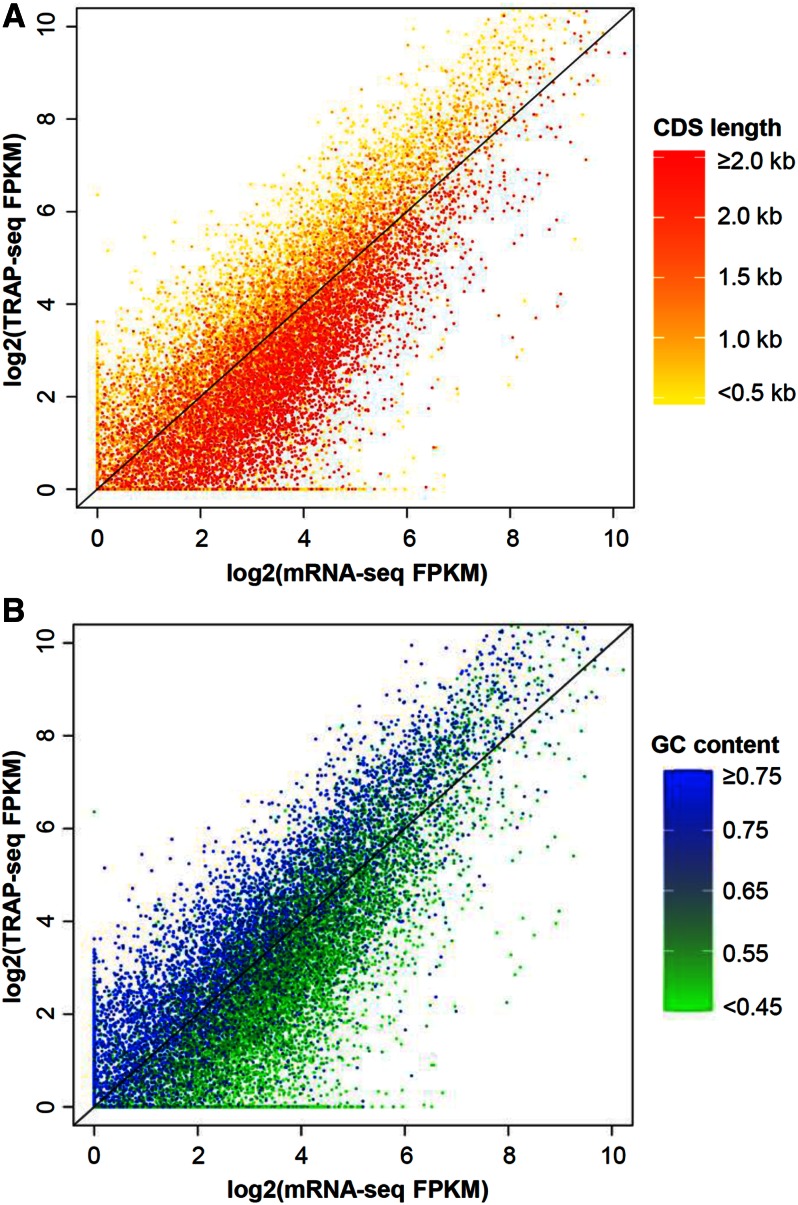
Plot of expression abundance at transcription level (mRNA-seq) *vs.* translation level (TRAP-seq) in relation to gene length (A) and GC content (B) of genes expressed in shoots. CDS, coding sequence; FPKM, fragments per kilobase per exon model per million mapped reads; mRNA-seq, messenger RNA sequencing; TRAP-seq, translating ribosome affinity purification followed by mRNA-sequencing.

In rice, the length of gene CDS varies substantially with a median value of 1095 bp (Table 3 and Table S5). To test whether CDS length is related to translatome enrichment, the level of mRNAs associated with ribosomes (TRAP-seq FPKM) was plotted against total mRNA abundance (mRNA-seq FPKM) for rice genes with different length of CDS. As shown in Figure 2A, genes with a large CDS tend to have a relatively low TRAP-seq level. When the CDS is shorter than 500 bp, the median value of TEI is about 2 (Figure S3). When the CDS length is longer than 1500 bp, the median value of TEI is about 0.5 (Figure S3). The difference between each group is significant (*P* < 0.0001, KS test). A similar trend in TEI with CDS length was observed in calli and young panicles (Figure S2 and Figure S3). These results are consistent with previous observations that short genes are associated with higher translation levels in other organisms (Arava *et al.* 2003; Jiao and Meyerowitz 2010; Lackner *et al.* 2007; Lei *et al.* 2015; Shah *et al.* 2013). It is feasible that, when the number of initiation factors or ribosomes is limiting and the two termini of a long transcript are further apart, the first initiation event and/or the efficiency of ribosome recycling and reinitiation is reduced and therefore fewer transcripts are translated.

**Table 3:** The effect of length and GC content of different portions of transcripts on TEI

Feature		5′ UTR (bp)*^a^*	CDS (bp)	3′ UTR (bp)
Size (bp)				
Mean Median Correlation		215	1234	433
		147	1095	354
		Negative	Negative	Optimal size 200–400 bp
GC content (%)				
Mean Median Correlation		58.3	56.1	39.3
		59.2	54.0	39.1
		Weak negative	Positive	Weak positive

UTR, untranslated region; CDS, coding sequence; TEI, translatome enrichment index.

a Only genes with both UTRs meeting size criterion (see *Materials and Methods* for details) and with TEI in at least one tissue were considered for calculation of mean and medium values.

As described in the introduction, the GC content of transcripts in grasses has a bimodal distribution (Figure S4) with the high GC fraction largely absent from dicot genomes. As shown in Figure 2B and Figure S2, the TEI value is positively correlated with GC content in all tissues. We then divided the rice genes into four groups (GC < 50%, 50–60%, 60–70%, and ≥ 70%) and examined the TEI. The average TEI of the highest GC group (≥70%) is nearly two whereas that for the lowest GC group is about 0.5 (*P* < 0.0001, KS test) (Figure S3). The preferential association of ribosomes with GC-rich genes is consistent with the results from a recent study in maize (Lei *et al.* 2015). Since the correlation between GC content and TEI is only reported in grasses, and GC-rich genes are often associated with small size (Guo *et al.* 2007), it was reasoned whether the elevated TEI of GC-rich genes is due to their small size. To this end, we divided rice genes into six groups with increasing CDS size and calculated the correlation between CDS size and GC content in each group. The result showed that the relationship between CDS length and GC content varies among different size ranges (Table S6 and Table S7). When the CDS length is between 1100 and 1400 bp, no significant correlation between CDS length and GC content was observed (Table S6, *P* = 0.33). Nevertheless, significant positive correlation (*P* < 2.2e−^16^) between GC content and TEI was still observed, suggesting that CDS length and GC content played independent roles in translation.

The high TEI associated with increased GC content suggests that the evolution of GC-rich genes in grasses may be associated with their increased translational activity. However, high GC transcripts are often associated with stronger secondary structure that may hinder the elongation process and/or cause ribosome stalling or pausing because the GC content variation is correlated with distinctions in codon usage bias (Chen *et al.* 2004; Fennoy and Bailey-Serres 1993; Kanaya *et al.* 2001; Knight *et al.* 2001), thereby resulting in a higher TEI. In a previous study, it was shown that GC-poor and GC-rich genes are subject to differential constraints in codon usage (Mukhopadhyay *et al.* 2007). The codon usage in GC-poor genes is largely selected for tRNA gene abundance; whereas that in GC-rich genes is selected both by tRNA gene abundance and by stable secondary structure (Mukhopadhyay *et al.* 2007). Accordingly, the selection for secondary structure in GC-rich genes could be due to its benefit in association with ribosomes. In mammalian cells, high GC content is associated with enhanced transcription activity (Kudla *et al.* 2006) and our analysis indicates that high GC content may also favor ribosome association of relevant transcripts. Application of the ribosome footprint profiling (Ribo-seq) method, which maps individual ribosomes along transcripts, could provide further insight into the underlying mechanisms associated with the higher TEI of GC-rich mRNAs.

As mentioned above, about one third of the “expressed proteins” or “hypothetical proteins” are likely translated. In general, the CDS of those “expressed proteins” or “hypothetical proteins” is much smaller than that of genes with known function (median 615 *vs.* 1179 bp, Table S8), and they are also more GC-rich (57 *vs.* 53%). The shorter CDS and higher GC content result in a higher TEI (1.14 *vs.* 0.88) compared to genes with known function. As a result, the transcripts derived from “expressed proteins” or “hypothetical proteins” are more enriched on ribosomes.

### Translatome enrichment for genes with different expression breadth and abundance

In yeast, shorter genes are more efficiently translated (Arava *et al.* 2003; Lackner *et al.* 2007), which was thought to be a consequence associated with the shortened length of constitutively expressed housekeeping genes (Hurowitz and Brown 2003; Shah *et al.* 2013). As such, it minimizes the possibility that transcript length itself may have a mechanistic role in translational regulation. To test whether this is the case in rice, we examined the translatome enrichment of constitutively expressed genes and putative housekeeping genes. To this end, 12 mRNA-seq datasets (Table S3) obtained from the NCBI SRA database along with the three mRNA-seq samples generated from this study (15 total mRNA-seq samples) were used to determine expression breadth. To annotate a gene as expressed, a FPKM of ≥ 1 in at least one tissue was required. With this criterion, three groups of genes were generated. One group includes genes expressed in 1–7 samples, the second group includes genes expressed in 8–14 samples, and the third group includes genes expressed in all 15 samples (constitutively expressed genes).

**Figure 3:**
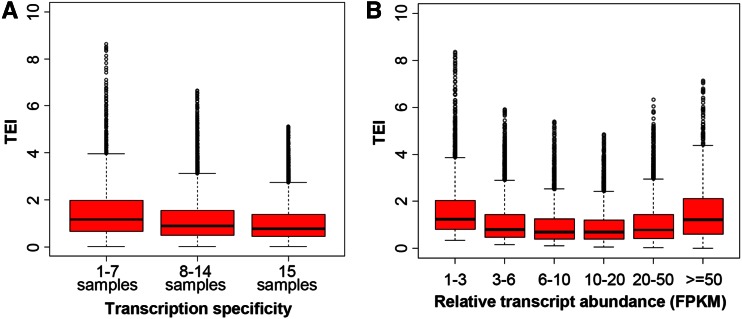
Distribution of translatome enrichment index (TEI) by transcription specificity and transcription level in shoots. (A) TEI of genes with transcripts detected across 15 mRNA-seq (messenger RNA sequencing) samples. (B) TEI of genes with transcript abundance variation as estimated by fragments per kilobase of exon per million mapped reads (FPKM). Genes with extremely high TEIs (1% of the genes in each group) were not shown.

As shown in Figure 3A, there is a minor yet negative correlation between expression breadth and TEI, with constitutively expressed genes having slightly lower TEI than other genes. This is consistent with the fact that the CDS of constitutively expressed genes are slightly longer and less GC-rich than other genes (Figure S5). We further examined the TEI of 23 known reference (housekeeping) genes (Narsai *et al.* 2010). The median value of TEI for those genes ranges from 0.98 to 1.05 in three tissues, which is comparable with that of other genes (0.93) (Table S9). The median length of the reference housekeeping genes is similar to that of other genes (1071 and 1095 bp, respectively, Table 3 and Table S9). Taken together, the high TEI value for short genes in rice is not due to the enrichment of constitutively expressed housekeeping genes, suggesting that there is a mechanistic role of a small CDS size in translation. Moreover, there is no evidence that housekeeping genes are preferentially translated in rice. If a high TEI value does imply a high amount of protein produced per transcript, the low transcript abundance of short GC-rich genes, such as the Poaceae lineage-specific genes (Campbell *et al.* 2007), maybe compensated by the higher translation efficiency, at least to some degree.

To explore the relationship between transcript level and translatome enrichment, genes were divided into six groups based on their expression abundance, with each group containing a similar number of genes (Table S4). As shown in Figure 3B, the TEI and expression abundance (relative steady-state transcript abundance) demonstrate a quadratic relationship, *i.e.*, the TEI is higher for the genes with the highest or lowest expression abundance, whereas the genes with intermediate expression abundance have the lowest TEI; the differences between the two extreme groups and other intermediate groups are significant (*P* < 0.0001, KS test). To understand the underlying mechanism of this relationship, the GC content and CDS length of each group were examined (Figure S5). Interestingly, the GC content of genes shows a similar quadratic relationship, *i.e.*, the genes with the lowest and highest transcription level have higher GC content than the genes with intermediate transcription abundance (Figure S5). Moreover, the length of CDS has a negative quadratic relationship with expression abundance, which suggests that genes with the highest and lowest transcription levels are associated with a smaller CDS. As a result, the nonlinear relationship between TEI and expression abundance can be largely explained by the GC content and CDS length of the relevant transcripts.

### Regulation of alternatively spliced transcripts at the translational level

When pre-mRNAs from genes with multiple exons are processed, a single transcript could be alternatively spliced into multiple isoforms. In humans, the majority of multi-exon genes have alternative spliced transcript isoforms (Pan *et al.* 2008; Wang *et al.* 2008), which allow the generation of multiple protein isoforms from a single gene. In plants, isoforms are not as prevalent as in mammals, yet they still play important roles in protein diversity. Previous studies in rice indicate that the majority of alternative splice forms result from the retention of individual introns (Campbell *et al.* 2006; E *et al.* 2013; Wang and Brendel 2006). Among the 16,809 isoforms annotated in MSU Release 7, 8164 isoform pairs were SI *vs.* RI, referring to isoforms of genes with spliced introns and retained introns, respectively (Campbell *et al.* 2006). Among them, a small subset are both transcribed and translated, with calli containing the highest number of expressed SI/RI pairs (732 pairs, Table S10 and Table S11). Whereas the median TEI of the SI isoform is comparable to other genes, the median TEI for RI isoforms is much lower than that of SI isoforms (Table S10), suggesting that the RI may have a negative impact on translation or that the transcripts with RIs may be inaccessible to ribosomes due to nuclear localization or cytoplasmic sequestration (Lavut and Raveh 2012).

**Figure 4:**
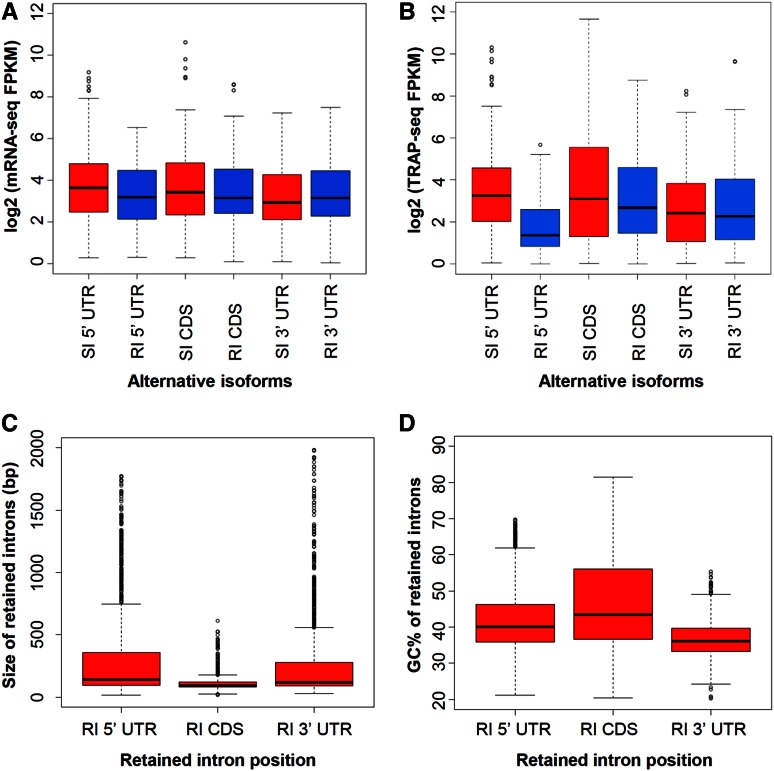
The impact of retained intron on transcription and translation of the relevant transcripts in shoots. (A) Expression abundance at transcription level (log2 FPKM of mRNA-seq) of isoforms with or without retained intron in different portions (5′ UTR, CDS, and 3′ UTR) of genes. (B) Expression abundance at translation level (log2 FPKM of TRAP-seq) of isoforms with a retained intron in different portions of genes. (C) Size of the retained introns. (D) GC content of the retained introns. Extreme values (top 1%) in each group were not shown. CDS, coding sequence; FPKM, fragments per kilobase per exon model per million mapped reads; mRNA-seq, messenger RNA sequencing; RI, retained intron; TRAP-seq, translating ribosome affinity purification followed by mRNA-sequencing; UTR, untranslated region.

The RIs were grouped based on their location in the mRNA, including those in the 5′ UTR, CDS, and 3′ UTR. The abundance of the RI transcript isoform, as reflected by the median FPKM value through mRNA-seq, is very similar to that of their SI counterpart (Figure 4). Nevertheless, when the RI is in a 5′ UTR, the TRAP-seq level of the RI isoforms is substantially lower than that of the corresponding SI isoforms (*P* < 0.0003, KS test) and the trend is similar in all three tissues (Figure 4, Figure S6 and Table S10). For RIs in the CDS and 3′ UTR, the TRAP-seq level of RI isoforms is slightly lower than that of the SI isoform, but the difference is not significant (*P* > 0.3, KS test). It should be noted that the lower TRAP-seq level is unlikely due to nonsense-mediated mRNA decay (NMD) (Kalyna *et al.* 2012). This is because NMD involves a functional 80S ribosome and therefore occurs after the initiation of translation (Schweingruber *et al.* 2013), so a more substantial reduction of transcript abundance is expected but this is not the case here.

To test whether the effect of the RI in different portions of the transcripts was due to the size and/or GC content of the RI, the relevant intron sequences were characterized. The median size of RIs is < 150 bp, much smaller than the median intron size of 418 bp in rice, suggesting that small introns are more likely to be retained (Figure 4). The size of the RI in the 5′ UTR is slightly larger than that in the 3′ UTR (Figure 4). The RIs in the CDS are the smallest, with a median value < 100 bp. As for GC content, the RIs in the CDS are associated with the highest GC content (Figure 4), followed by that in the 5′ UTR, with the RIs in the 3′ UTR having the lowest GC content. Not surprisingly, the GC content of the RIs is lower than the corresponding genomic average GC content of the 5′ UTR, CDS, and 3′ UTR. Moreover, the difference in GC content between RI in the 5′ UTR and other 5′ UTR sequences is the most significant (40 *vs.* 57%, Figure 4 and Table 3). As a result, the dramatically lower TRAP-seq level caused by RI in 5′ UTRs may be due to its slightly larger size and/or its lower GC content than average 5′ UTRs (see below).

If RIs in the CDS leads to the generation of different protein isoforms, the evolutionary advantage for RIs in UTRs is unclear. Emerging evidence suggests that RIs followed by cytoplasmic splicing provide a newly recognized regulation of gene expression, especially for developmentally controlled genes. In the fern *Marsilea vestita*, large amounts of RI-containing transcripts are stored and later spliced in the microspore during spermatogenesis (Boothby *et al.* 2013). In this way, proteins can be rapidly generated without the need for *de novo* transcription. These RI transcripts are hypothesized to be “sentinel RNAs” (Buckley *et al.* 2014). Our analysis suggests that, for some rice genes, the RI forms are transcribed similarly to their corresponding SI forms but are underrepresented on ribosomes, suggesting that “sentinel RNAs” may also apply to those rice genes. To determine what types of genes are more likely to show intron retention, the GO terms were assigned to the SI/RI isoforms and compared with that to all genes. The genes that are most enriched (χ^2^ test, *P* < 0.001) in SI/RI isoforms are those involved in the response to abiotic stimuli, followed by those in postembryonic development, growth, and the generation of precursor metabolites and energy (χ^2^ test, *P* < 0.05, Table S12). Given that the SI/RI isoforms are enriched in genes involved in the response to abiotic stimuli and some development processes, it is conceivable that the splicing and translation of the preexisting RI transcripts would allow a rapid response to abiotic stimuli as well as important developmental signals.

### The role of UTRs in translation

**Figure 5:**
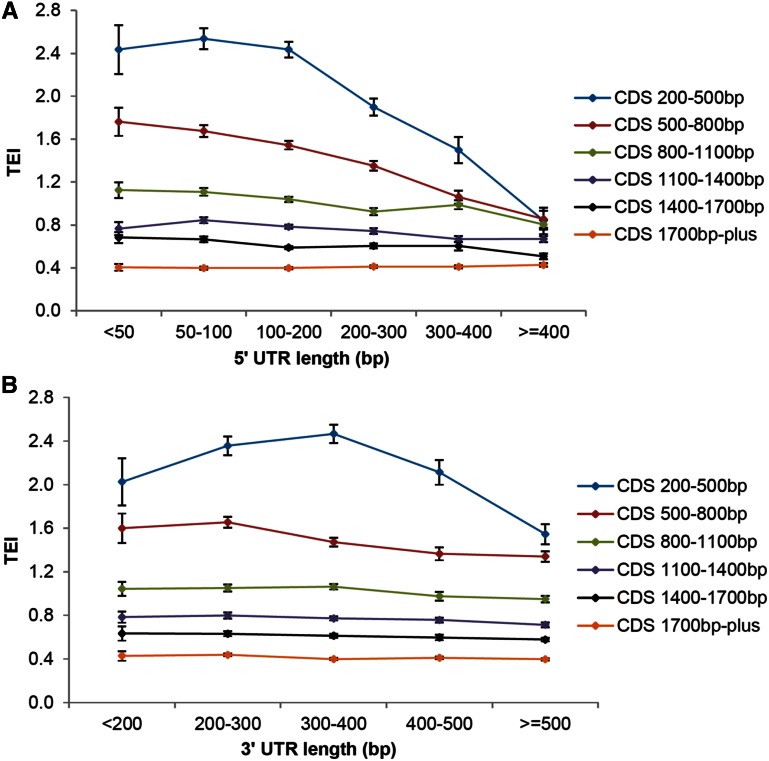
Correlation between translatome enrichment index (TEI) and size of untranslated regions (UTRs) in shoots. (A) Variation of TEI with length of 5′ UTR. (B) Variation of TEI with length of 3′ UTR. Error bars correspond to 2 × SE. CDS, coding sequence.

The differential TEI between RI/SI isoform pairs allows further dissection of factors involved in translation. To explore the roles of the UTRs in translation, we extended our analysis to all nontransposon genes with annotated 5′ and 3′ UTRs. To minimize the effect of CDS, we divided the genes into different groups based on CDS length and examined the effect of UTR length within each group. In general, there is a negative correlation between the size of 5′ UTRs and TEI (coefficient < 0, *P* < 10^−7^ for all gene groups except the group with CDS > 1700 bp) (Figure 5). The effect is more dramatic among genes with CDS lengths smaller than 800 bp (coefficient < 0, *P* < 10^−15^), which includes about one-third of all translated transcripts. For genes with CDS shorter than 800 bp, if the 5′ UTR is smaller than 200 bp, the TEI is significantly higher than for genes with a 5′ UTR longer than 200 bp (Figure 5, *P* < 0.0001, KS test). Particularly, when the 5′ UTR is longer than 400 bp, all genes have a low TEI (<1) regardless of their CDS length (Figure 5A), suggesting that an extended 5′ UTR could be detrimental to translation. The same trend is observed in calli and panicles (Figure S7). For the 3′ UTR, there seems to be an optimal size for TEI, when the CDS is smaller than 500 bp (Figure 5) (*P* < 10^−12^, quadratic regression). The same trend is observed in calli but not in panicles (Figure S7). Overall, the size effect of the 5′ UTR on TEI is more substantial and consistent than that of the 3′ UTR.

If the GC content of the UTR is examined in relationship to the TEI, the TEI value declines with increasing GC content in the 5′ UTR, if both the CDS and the 5′ UTR are small (CDS < 500 bp and 5′ UTR < 200 bp; Figure S8). This is consistent with the observation that high GC content in a 5′ UTR region significantly reduces ribosome loading (Kawaguchi and Bailey-Serres 2005). In contrast, TEI values increase slightly with increasing GC content of 3′ UTRs (Figure S8), when CDS is <500 bp. However, compared with the effect of size of the UTR, the correlation between GC content of UTRs and TEI is rather weak and the difference between most data points is not significant (*P* > 0.05, KS test). Based on results from this analysis, the impact of RI in a 5′ UTR on translation is most likely due to its effect of increased size of the 5′ UTR, not due to the influence on GC content *per se*. Taken together, each portion of the transcript (5′ UTR, CDS, and 3′ UTR) plays a role in translation, depending on length, GC content, and possibly other sequence features or elements within these regions. The impact of UTRs is most significant for small genes. A summary of the correlation between these features and TEI is given in Table 3.

Translation in eukaryotes is initialized by the recruitment of the 40S ribosome preinitiation complex and then the 60S ribosomal subunit to the 5′ end of the transcript, followed by subunit joining and ribosome translocation from codon-to-codon as elongation proceeds (Araujo *et al.* 2012; Browning and Bailey-Serres 2015). It is conceivable that a short 5′ UTR is favorable for translation over a long 5′ UTR because of a lower energy requirement for unwinding the 5′ UTR during the preinitiation complex scanning process. Many 5′ UTRs also harbor one or more uORFs or upstream start codons (uAUGs), which often reduce frequency of initiation of translation of the main open ORF encoding the gene product. A large fraction of uORFs are indeed translated in *Arabidopsis* (Juntawong *et al.* 2014). Based on mammalian studies, some of the peptides encoded by uORFs might be functional (Ji *et al.* 2015), and translation of uORFs seems to play key roles in the integrated stress response (Starck *et al.* 2016). However, when a uORF is translated, the ribosome reinitiation for the downstream main ORF is inefficient, which causes translational suppression of the main ORF (Roy and von Arnim 2013). Since longer 5′ UTRs more likely contain uORFs or uAUGs, it might be anticipated that longer 5′ UTRs are associated with lower TEI. It is worthy of mention that RPL18 is a component of the 60S ribosomal subunit, which only assembles on the mRNA once the start codon AUG is identified by the scanning preinitiation complex composed of the 40S subunit and initiation factors. As a result, only mRNAs that are associated with at least one 80S ribosome can be obtained by TRAP and hence are detected in this study. Moreover, translation is reduced by stable secondary structures present in the 5′ UTR (Araujo *et al.* 2012). This is likely to explain the minor negative impact of GC content of the 5′ UTR on TEI because GC-rich sequences are more likely to form thermodynamically stable structures (Kawaguchi and Bailey-Serres 2005; Nguyen and Southern 2000).

### Translatome enrichment of genes in different plant lineages

**Figure 6:**
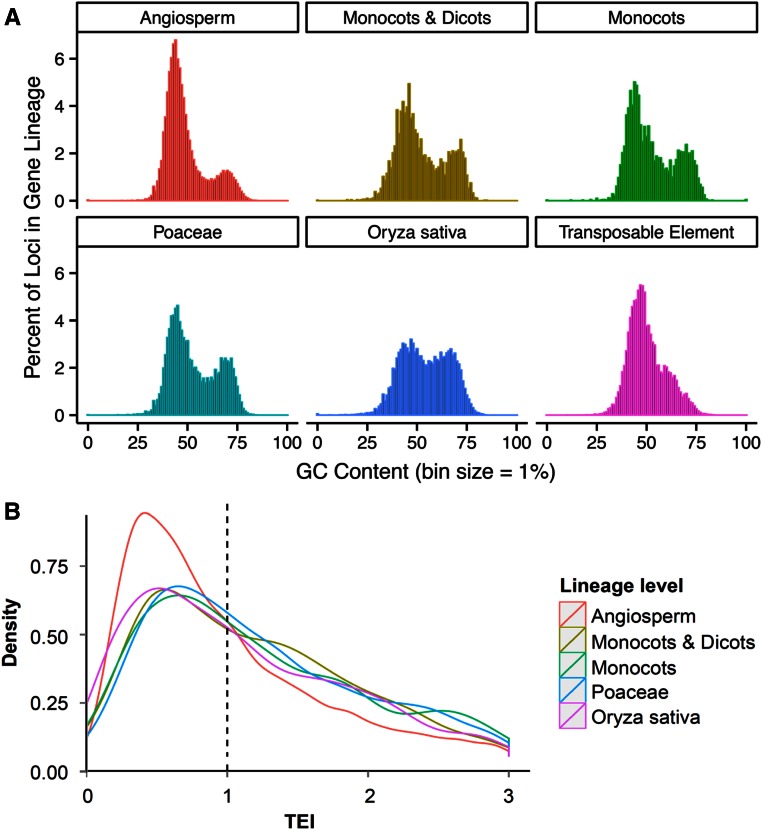
GC content and translatome enrichment index (TEI) of rice genes based on their evolutionary origin. (A) GC content of rice non-TE genes based on orthologous clustering with predicted proteomes from 12 angiosperm species. (B) TEI of nontransposon genes in rice shoots based on orthologous clustering with predicted proteomes from 12 angiosperm species.

To consider GC content and TEI in the context of the evolutionary origin of rice genes, nontransposon genes were compared with genes from 12 other flowering plants and assigned to nonoverlapping plant lineages based on their placement within orthologous gene clusters generated with OrthoMCL. Genes in a lineage may have arisen in a specific evolutionary time frame. Classifications included genes conserved in all angiosperms (most ancestral), genes conserved between monocots and dicots (second ancestral), monocot-specific (third ancestral), Poaceae-specific (fourth ancestral), and rice (*O. sativa*)-specific genes (most recent) (see Table S13 for list of rice genes in each lineage). When the GC content of the CDS of gene transcripts of each lineage was profiled (Figure 6A), genes conserved in all angiosperms had the lowest GC content, and the most recent genes (rice-specific) were associated with the highest fraction of GC-rich genes, indicating that the GC-rich module has been progressively enriched in the evolutionary history of rice. Notably, the GC-rich module is largely absent for transposable elements (Figure 6), which explains why transposons have rather low TEIs compared to nontransposon genes. The fluctuation of the GC content among different gene lineages may reflect the variation in activity of different gene duplication mechanisms. For example, it is known that Pack-MULEs, which refer to *Mutator*-like transposable elements carrying gene or gene fragments, have been highly active recently in the rice genome and they specifically duplicate GC-rich gene fragments (Jiang *et al.* 2011). As a result, they may contribute to the high GC content of rice-specific genes. It is worthy of mention that, due to their ability to duplicate genes, Pack-MULEs have distinct GC content compared to other transposable elements (Ferguson *et al.* 2013).

The TEIs of genes in these different lineage groups were examined (Figure 6B, Figure S9, and Table S13). In each lineage and in all three tissues, there is a broad range of TEIs ranging from near “0” to > 3. Consistent with our analyses based on GC content and the bimodal nature and enrichment of high GC content genes in rice, genes that are conserved among all angiosperms and those with overall lower GC content had the lowest fraction of genes with a TEI ≥ 1. This is also reflected in TEI distribution profiles; rice genes that are conserved in all angiosperms have a more discrete TEI distribution with a clear peak TEI value of ≤1, whereas all other rice genes have a broader TEI distribution regardless of whether they are restricted to monocots and dicots, monocot-specific, Poaceae-specific, or *O. sativa*-specific. Overall, these data suggest the evolutionarily recent genes in rice are more likely to be associated with ribosomes than core genes present in all angiosperms. Whether this is due to unique functions that arose through evolution or is a consequence of higher GC content found in rice and the Poaceae remains to be determined.

### Genes involved in distinct biological processes are differentially enriched on ribosomes

The dramatic difference in TEI of different genes suggests that the translation process is tightly regulated; however, the functional relevance of such regulation is unclear. To this end, we examined the translatome enrichment of genes involved in different biological processes according to GO terms. The TEI values of genes for different processes vary significantly, with mean values ranging from 0.53 to 1.78 in shoots (Table 4 and Table S14). Interestingly, the process that is associated with the highest TEI in shoots is translation itself, followed by DNA metabolic process and photosynthesis (Table 4). Other processes associated with high TEIs are those involved in the generation of precursor metabolites and energy, cellular homeostasis, secondary metabolic processes, and the response to stress including both abiotic and biotic stresses. Most of these processes seem to be critical for the survival of the plants. The process associated with the lowest TEI is related to “regulation of gene expression, epigenetic,” followed by those involved in carbohydrate metabolism, lipid metabolism, growth, cell cycle and differentiation, protein modification, and multicellular organismal development (Table 4), which might be less critical for survival. Importantly, the trend observed in shoots is also seen in calli and panicles except that in calli, photosynthesis is not among the top three processes (Table S14). This is understandable since photosynthesis is not active in calli, suggesting that translation regulation may be dependent on cellular energy status. In general, the genes involved in processes with low TEIs are associated with moderate expression abundance (mRNA-seq FPKM values ranging from 17 to 46), while genes in processes with high TEIs could be associated with very high expression abundance (mRNA-seq FPKM values ranging from 28 to 449) (Table 4).

**Table 4:** Ten biological processes in which genes are associated with the highest and lowest TEI in rice shoots

GO Term (Process)	TEI	mRNA-seq (FPKM)*^a^*	TRAP-seq (FPKM)	CDS size (bp)*^b^*	5′ UTR Length*^b^*	CDS GC Content (%)*^b^*
GO processes with high TEI						
Translation	1.78	69.6	173.6	956 (−59)	141 (34)	54.5 (8.6)
DNA metabolic process	1.47	44.2	87.8	1183 (−133)	175 (47)	53.6 (9.0)
Photosynthesis	1.47	430.9	986.3	1019 (−46)	138 (30)	59.6 (12.9)
Generation of precursor metabolites and energy	1.36	190.3	387.5	1174 (−33)	168 (54)	57.1 (10.8)
Cellular homeostasis	1.34	68.1	248.4	1207 (−36)	201 (60)	55.9 (10.5)
Response to stress	1.31	48.0	67.9	1305 (−21)	200 (59)	56.5 (11.0)
Response to abiotic stimulus	1.29	77.6	126.8	1294 (−17)	205 (59)	57.4 (11.5)
Secondary metabolic process	1.27	26.4	32.3	1215 (−59)	160 (44)	61.0 (15.7)
Biological process	1.22	27.6	37.0	1171 (12)	206 (60)	55.5 (10.5)
Response to biotic stimulus	1.19	47.4	56.9	1385 (−86)	209 (66)	56.9 (11.1)
Mean	1.37	103.0	220.4	1191 (−48)	180 (51)	56.8 (11.2)
GO processes with low TEI						
Regulation of gene expression, epigenetic	0.53	16.9	10.2	2084 (−79)	207 (58)	47.7 (3.2)
Carbohydrate metabolic process	0.73	43.3	42.6	1577 (−44)	222 (73)	54.8 (9.8)
Lipid metabolic process	0.74	28.8	20.7	1383 (−32)	208 (54)	54.6 (9.9)
Cell differentiation	0.86	20.8	18.1	1589 (−82)	238 (51)	54.5 (9.2)
Protein modification process	0.86	28.8	42.1	1496 (−37)	270 (81)	52.3 (7.7)
Cell cycle	0.87	16.7	13.7	1492 (−230)	247 (78)	51.0 (6.9)
Catabolic process	0.90	44.5	45.2	1502 (−84)	220 (77)	52.6 (7.5)
Cell growth	0.91	19.9	17.4	1560 (−92)	191 (36)	55.1 (9.5)
Growth	0.92	22.5	21.2	1605 (−32)	245 (102)	53.6 (8.2)
Multicellular organismal development	0.92	21.3	18.1	1556 (−91)	235 (63)	54.9 (9.8)
Mean	0.82	26.3	24.9	1584 (−80)	228 (67)	53.1 (8.2)
Genome-wide mean*^c^*	1.21	33.4	56.0	1208	212	55.6
Difference with high TEI processes	−0.55	−76.7	−195.5	394 (−33)	48 (16)	−3.7 (−3.0)
% Variation*^d^*	67.10	291.6	785.1	33.1	l26.7	7.0

GO, gene ontology; TEI, translatome enrichment index; mRNA-seq, messenger RNA sequencing; FPKM, fragments per kilobase per exon model per million mapped reads; TRAP-seq, Translating Ribosome Affinity Purification followed by mRNA-sequencing; CDS, coding sequence; UTR, untranslated region.

a Only processes with 40 or more genes are considered. The values are mean values in each process.

b Numbers in parentheses indicate difference compared with the corresponding *Arabidopsis* processes.

c Genome-wide mean of 5′ UTR is derived from nontransposon genes with TEI in shoots and UTRs ≥ minimum (20 bp for 5′ UTR and 110 bp for 3′ UTR).

d %Variation equals difference divided by the lower values of the two means, then times 100.

Consistent with the analyses above, a large portion of the variation in TEI of genes in different processes can be attributed to the properties of the transcripts. All features (TEI, mRNA-seq level, TRAP-seq level, CDS size, 5′ UTR length, and GC content of CDS) described in Table 4 are significantly different between the genes with high TEIs and low TEIs (KS test, *P* < 0.0001). In general, genes with high TEIs have short CDS, a short 5′ UTR, and high GC content in coding regions (Table 4). Conversely, genes involved in the processes with low TEIs are longer than average (both CDS and 5′ UTR) and the GC content is lower than average (Table 4). Finally, the GC content of 5′ UTRs is not significantly different between the high TEI and low TEI groups (59.0 *vs.* 59.5, KS test, *P* = 0.56) (Table S14). The GC content of 3′ UTRs is only slightly different (39.2 *vs.* 39.7 KS test, *P* = 0.06). Again, this is consistent with the notion that the GC content of UTRs is associated with a minor influence on translation in rice.

### Relationship between codon usage bias, recombination rate, and TEI

As discussed above, the preferential association of GC-rich transcripts with ribosomes could be due to the physical property of the transcript, such as secondary structure. Nevertheless, it could be also due to preference on codon usage, which is often linked to tRNA abundance. It has been well established that codon usage differs among and within organisms, which is referred to as codon usage bias (Behura and Severson 2013). Studies showed that highly expressed genes exhibited high codon bias in *Escherichia coli*, *Caenorhabditis*, *Drosophila*, and *Arabidopsis* (Duret and Mouchiroud 1999; Sharp and Li 1986). To determine whether the codon usage bias is associated with differential ribosomal enrichment, the codon usage of genes with the extremely low (<0.2) and extremely high (≥3) TEIs were compared in shoots. As shown in Table S15, all 18 amino acids with multiple synonymous codons exhibited different codon usage frequency. Overall, genes with low TEIs showed a minor preference to codons with A/U at the third codon position whereas those with high TEIs had extremely significant bias toward G/C at the third codon position. Further dissection of codon usage revealed that base composition of the first codon position also plays a role toward the usage bias (Table S16). For all four pairs of synonymous codons differing only at the first codon position, genes with high TEIs showed significant preference to C than to A/U. The pair with the most dramatic difference is CUG *vs.* UUG for leucine, where the ratio of these two codons increased from 0.83 for low TEI genes to 4.75 for high TEI genes (Table S16). The impact of codon composition at the third codon position was also further investigated. In addition to the general preference for G/C for high TEI genes, the frequency varies between G and C (Table S16). For some pairs of synonymous codons, G is more preferred to C in high TEI genes (*e.g.*, the ratios between CCG and CCC for proline is 0.95 for low TEI genes and 1.77 for high TEI genes). Other pairs demonstrate the opposite trend (*e.g.*, the ratios between CUG and CUC for leucine declines from 1.16 for low TEI genes to 0.68 for high TEI genes). Different frequency is also observed for codons with A and U at the third codon position (Table S16). Among nine pairs of synonymous codons with A *vs.* U at the third codon position, five pairs showed significant usage bias between low and high TEI genes (*P* < 0.05). The codon discrimination between G and C as well as between A and U at the third codon position clearly suggests that codon usage plays a role in differential association of transcripts with ribosomes.

The significant correlation between GC content and TEI suggests that the emergence of GC-rich genes in grasses could be a result of selection for translation efficiency. However, other factors have been proposed to be responsible for the evolution of the GC content of genes. gBGC is a process that results in gene alleles with G/C at heterozygous sites being preferentially retained compared with alleles with A/T at heterozygous sites during recombination, which would lead to a high GC content at regions with high recombination rates. Recently, a large body of evidence suggested that gBGC is likely a major force in shaping chromosomal base compositions in both plants and animals (Duret and Galtier 2009; Pessia *et al.* 2012; Ressayre *et al.* 2015). To test if the GC content of genes is associated with the genetic recombination rate in rice, we used the local recombination rate from a previous study (Tian *et al.* 2009) and compared it with CDS GC content and TEI. A negligible correlation (correlation coefficient = 0.03, *P* < 10^−4^) was detected between the GC content of CDS and the recombination rate in rice, which was in contrast to the situation observed in *Gallus gallus*, where GC content was well correlated with recombination rate (correlation coefficient = 0.89, *P* < 10^−4^) (Pessia *et al.* 2012). This is consistent with the results from previous studies showing a lack of significant correlation between GC content and recombination rate in rice (Pessia *et al.* 2012; Tian *et al.* 2009). Moreover, the influence of recombination rate on TEI seems to be even more minor (correlation coefficient = 0.019801, *P* = 0.01526). Accordingly, it is unclear whether gBGC has played a major role in shaping the GC content of genes in rice. Nevertheless, it should be noted that the recombination map that is used in this study contains < 4000 markers, which is insufficient to distinguish the recombination rate of individual genes. Future studies with higher resolution recombination maps will facilitate our understanding of the contribution of gBGC to GC content evolution in rice. In addition, the contribution of selection on translation efficiency and gBGC to GC content variation could be complimentary instead of mutually exclusive. It is possible that GC-rich sequences were generated through gBGC and were retained because of their advantage in translation.

### Conservation and divergence of translational features in rice and Arabidopsis

To test whether the differential enrichment of transcripts on ribosomes is present in other plants, a comparative analysis was conducted using the data from *Arabidopsis* seedlings growing under standard conditions on MS media (Juntawong *et al.* 2014). To make the two datasets as comparable as possible, our analysis only included the *Arabidopsis* genes that correspond to genes used for GO term analysis in rice (see *Materials and Methods* for details). The relative order of TEI among genes in different biological processes is largely conserved between the seedlings of *Arabidopsis* and shoots of rice. For example, 9 of the top 10 processes with the highest TEI are shared in the two species, including photosynthesis, translation, generation of energy, cellular homeostasis, and responses to stresses (Table 4 and Table S17). Similarly, 7 of the 10 processes with the least enriched transcripts on ribosomes are shared between the two species (Tables 4 and Table S17), suggesting that divergent plants prioritize their biological processes similarly at the translational level.

**Figure 7:**
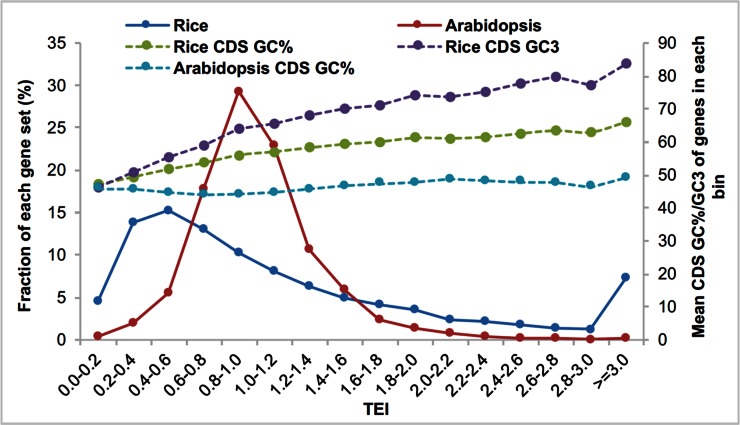
Fraction (%) of rice and *Arabidopsis* genes with different TEIs and their average GC and GC3 content of CDS. CDS, coding sequence; TEI, translatome enrichment index.

Although the relative order of transcript enrichment on ribosomes is largely conserved between *Arabidopsis* and rice, the degree of variation is quite different. In *Arabidopsis*, the mean TEI ranges from 0.91 to 1.14 (22% variation, Table S17) for different processes, whereas that for rice is from 0.53 to 1.78 (Tables 4 and Table S14), with over threefold variation. The variation in CDS size as well as in 5′ UTR length between high TEI and low TEI processes is rather comparable between rice and *Arabidopsis* (33 *vs.* 32%, 27 *vs.* 20%, Table 4 and Table S17). In contrast, the variation of GC content of CDS is considerably higher in rice than that in *Arabidopsis* (7.0 *vs.* 1.7%). Genome-wide, the TEI of genes in *Arabidopsis* demonstrates a nearly normal distribution, with a maximum fraction of genes around 1.0 (Figure 7). Most genes (over 80%) are associated with a TEI ranging from 0.6 to 1.4; only a small portion of the genes (∼2%) are associated with a TEI ≥ 2 (Figure 7). Furthermore, there is little variation in the GC content of CDS in different TEI bins in *Arabidopsis*. In contrast, distribution of the TEI in rice is widely dispersed. Less than 40% of the genes are associated with a TEI of 0.6–1.4, and 17% of the genes are associated with a TEI ≥ 2. Moreover, the GC content and GC3 content (GC content of the third codon position) of CDS of rice genes progressively increases as the TEI does (Figure 7). As a result, the variation of GC content of genes in rice could contribute to the more dramatic variation of TEIs observed in rice. It should be pointed out that the *Arabidopsis* and rice plants were not grown under exactly the same conditions and were not sampled at equivalent developmental stages. Nevertheless, highly similarly trends were observed among three distinct rice tissues, so it is unlikely that the dramatic difference in TEI distribution between *Arabidopsis* and rice is solely due to tissue or developmental specificity.

On average, the CDS of rice genes is about 10% more GC-rich than that in *Arabidopsis* (55.6 *vs.* 45.1%, Table 4 and Table S17), and GC-rich genes are associated with stronger codon usage bias (Fennoy and Bailey-Serres 1993). If the GC content of individual processes is examined, the increase of the GC content of rice genes (compared with that of *Arabidopsis*) in all the processes with low TEIs is less than the average value (Table 4). In contrast, genes in 8 out of the 10 GO categories with high TEIs are associated with a GC content increase equal to or higher than average (Table 4). The two exceptions are genes in translation and DNA metabolism; nevertheless, genes that encode proteins that function in translation have the smallest coding region in rice, and genes in DNA metabolism have experienced the largest reduction in CDS size (from *Arabidopsis* to rice) compared to other genes (Table 4). As a consequence, in most cases the high TEI in rice is correlated with increased GC content of CDS but could be due to the relatively small size of genes. Again, it should be noted that high TEI does not necessarily mean high protein abundance; however, if GC-rich or short mRNAs are more likely associated with ribosomes, it may increase the competition for GC-poor or long mRNAs to be translated due to limited availability of the translation machinery.

A strong example of such a relationship is the genes involved in “regulation of gene expression, epigenetic” (GO: 0040029) process. The genes in this process are associated with the largest and comparable mean CDS size in both *Arabidopsis* and rice (2163 and 2084 bp, respectively, Table 4 and Table S17). In *Arabidopsis*, the mean TEI of genes in this process is 0.92 whereas in rice it is 0.53 (Table 4 and Table S17). In rice, genes in all other GO categories have a substantially higher GC content (compared to their counterparts in *Arabidopsis*), ranging from 7 to 16% (Table 4). In contrast, the GC content difference between *Arabidopsis* and rice genes associated with epigenetic regulation of expression was only 3%. In addition, genes associated with this GO category had the lowest average GC content (47.7%) among all GO-associated rice genes (Table S14). The extremely low GC content of those genes is associated with the lowest mean TEI in rice and this was observed in all three rice tissues (mean TEI = 0.53, 0.60, 0.46 in shoots, calli, and panicles, respectively, Table S14). Since genes that function in epigenetic regulation are responsible for the regulation of transposable element activity, it raises the question whether the compromise in translation of these genes in rice has contributed to the high degree of amplification of transposable elements in rice compared to *Arabidopsis* (International Rice Genome Sequencing Project 2005; The Arabidopsis Genome Initiative 2000). Future analysis is required to test this hypothesis.

### Conclusions

In this study, we provide a genome-wide analysis of the translation status of genes in rice. Based on our analysis, it is clear that, for individual gene transcripts, the fraction that is ribosome-associated can vary dramatically. In rice, a large portion of the variation can be attributed to the length and the GC content of the coding region, suggesting that the evolution of GC-rich genes in monocots (especially in grasses) may be associated with an advantage in translation. In addition, UTRs, especially 5′ UTRs, play a critical role in translation. Small genes are preferentially associated with ribosomes; however, if the transcript has a 5′ UTR that is longer than 400 bp, the favorable effect of the short transcript is masked by the inhibitory effect of a long 5′ UTR.

Genes encoding proteins associated with different processes are differentially enriched on ribosomes. Genes involved in processes that are critical for plant survival, such as those related to photosynthesis, translation, and stresses responses, are more likely associated with ribosomes. Compared to corresponding translation status in *Arabidopsis*, mRNAs in rice have a more dramatic variation of TEI, which may result in differential regulation at the translational level that could be linked to phenotypic diversity. The wide variation of TEI in rice is correlated with more substantial variation of the GC content of genes, which is largely due to the emergence of GC-rich genes after the formation of angiosperms. Clearly, genes in different biological processes evolve differentially in terms of GC content, which may alter the dynamics of the relative translation efficiency of these processes. Since high TEI genes demonstrate an extraordinary bias toward GC-rich codons, it is likely that the genes in critical biological processes are more likely to retain AT to GC mutations. However, from this study, it is unclear whether AT to GC mutations are more frequently generated in some genes than in other genes, through processes such as gBGC. Future analysis with high resolution recombination maps will determine the role of gBGC in GC content evolution in rice. Despite the uncertainty of sources of GC biased mutation, we demonstrated that the low GC content of genes in epigenetic pathways in rice may be disadvantageous for their translation.

Future experimentation is required to test whether an enhanced TEI value is associated with greater protein production. If it is, factors that impact TEI can be utilized for future crop engineering. For example, more efficient translation of a target gene can potentially be achieved by using optimal (high frequency codons found within genes with high TEI) codons or minimizing the 5′ UTR. For genes with very short coding regions, a 3′ UTR with an appropriate length is beneficial for its translation. It should be noted that, in this study, the observations are made based on only three rice tissues, with two of them (callus and young panicle) composed of a mixture of rather divergent cells. Experiments with more tissues, especially those from more specialized organs, will reveal whether translational regulation is controlled in a tissue-specific fashion. In addition, all experiments conducted in this study are under standard growth conditions, and it will be intriguing to study the impact of environmental factors on translation such as the inhibition of translation initiation under hypoxia stress in *Arabidopsis* (Juntawong *et al.* 2014). Furthermore, it would be interesting to test where the RI transcript isoforms are located (nucleus or cytoplasm) and whether their relative abundance is reduced under stressed conditions.

## Supplementary Material

Supplemental material is available online at www.g3journal.org/lookup/suppl/doi:10.1534/g3.116.036020/-/DC1.
