# Supplementary figures and images for: Analysis of Ribosome-Associated mRNAs in Rice Reveals the Importance of Transcript Size and GC Content in Translation
Source: G3 (Bethesda). 2016 Nov 14;7(1):203–19. doi: 10.1534/g3.116.036020 (PMC5217110; doi:10.1534/g3.116.036020)

## Slide 1
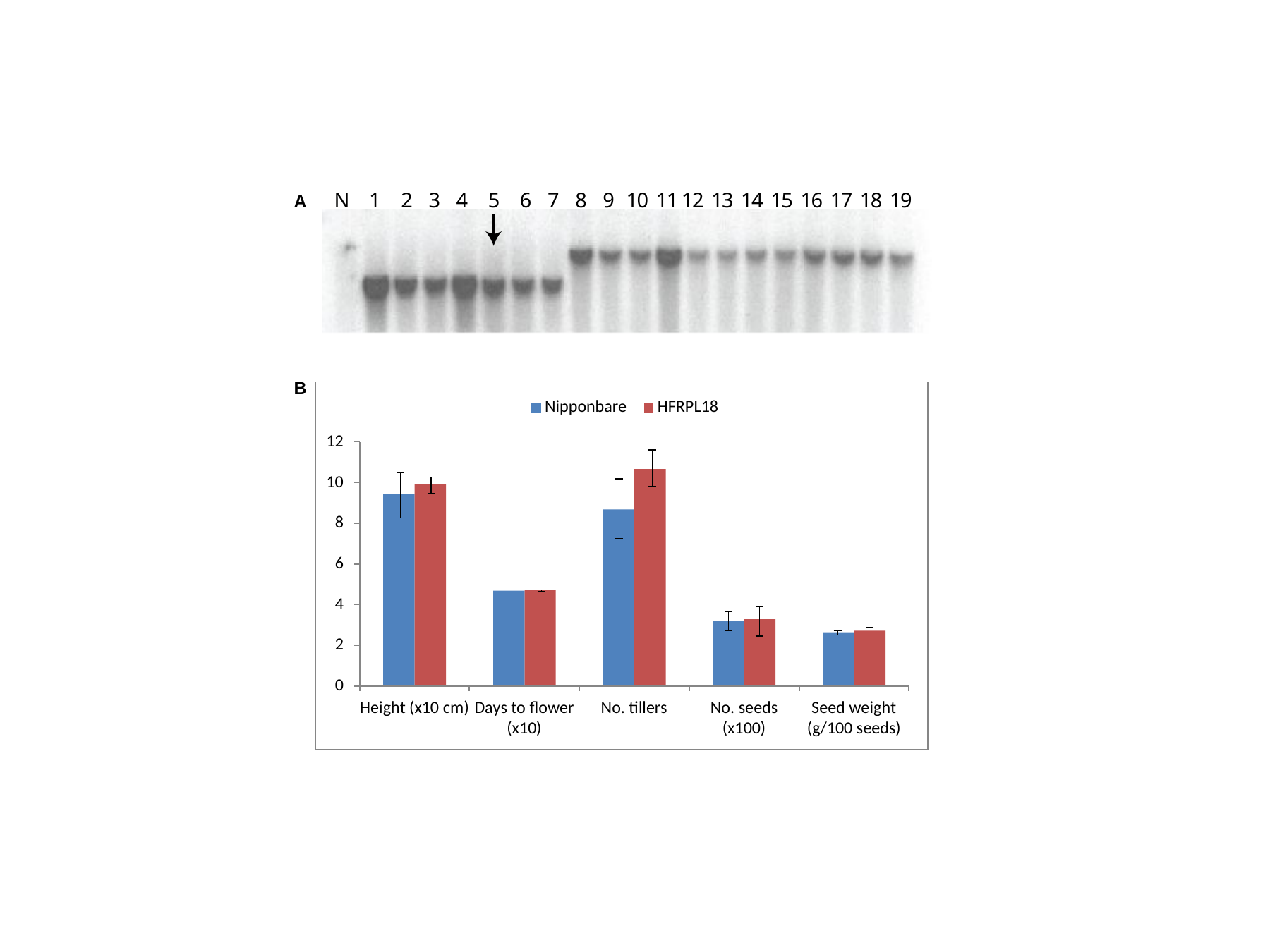

A
B

Supplement: Supplementary file 1 [file 203FigureS1.pptx]

## Slide 1
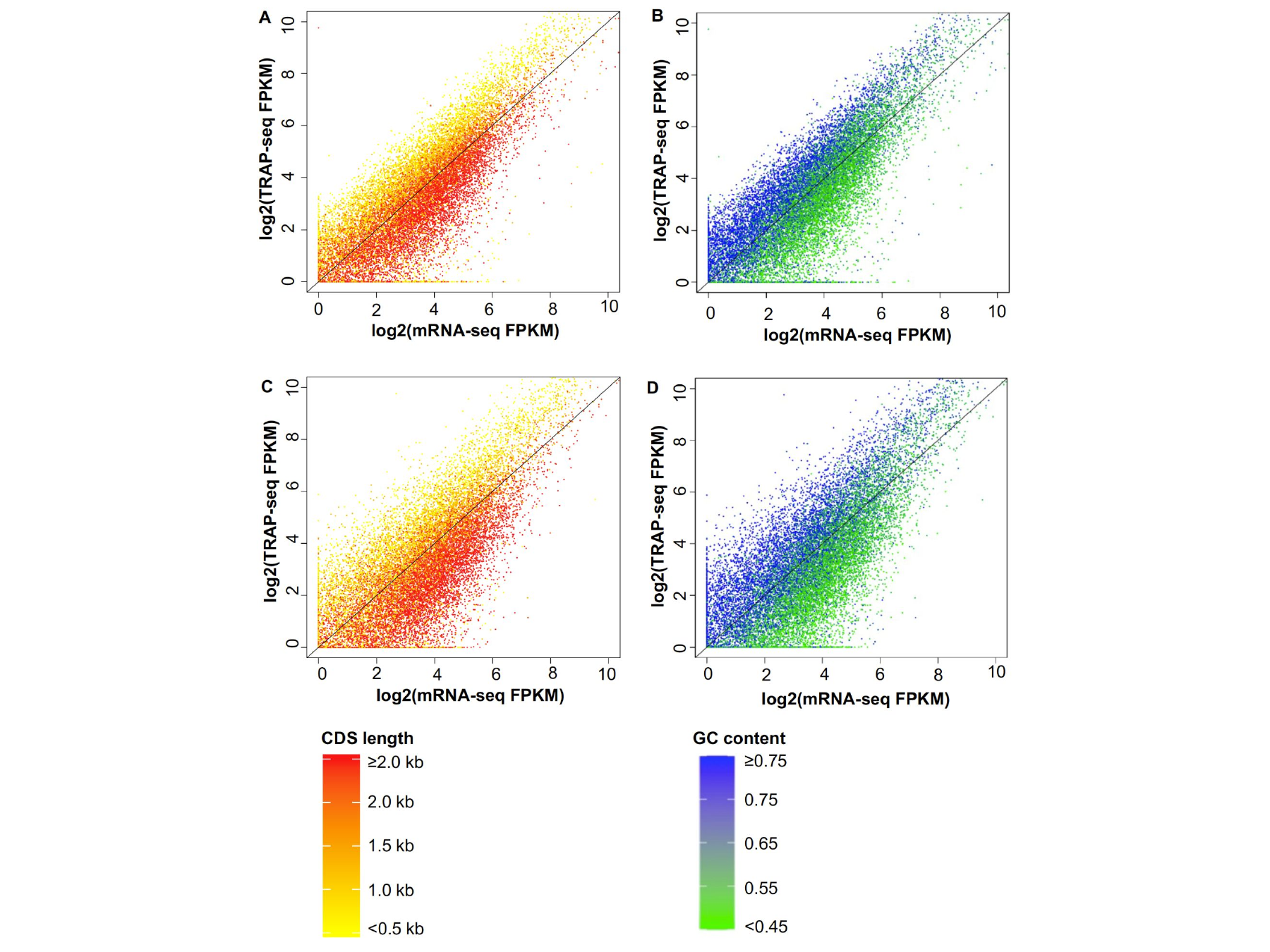

Supplement: Supplementary file 2 [file 203FigureS2.pptx]

## Slide 1
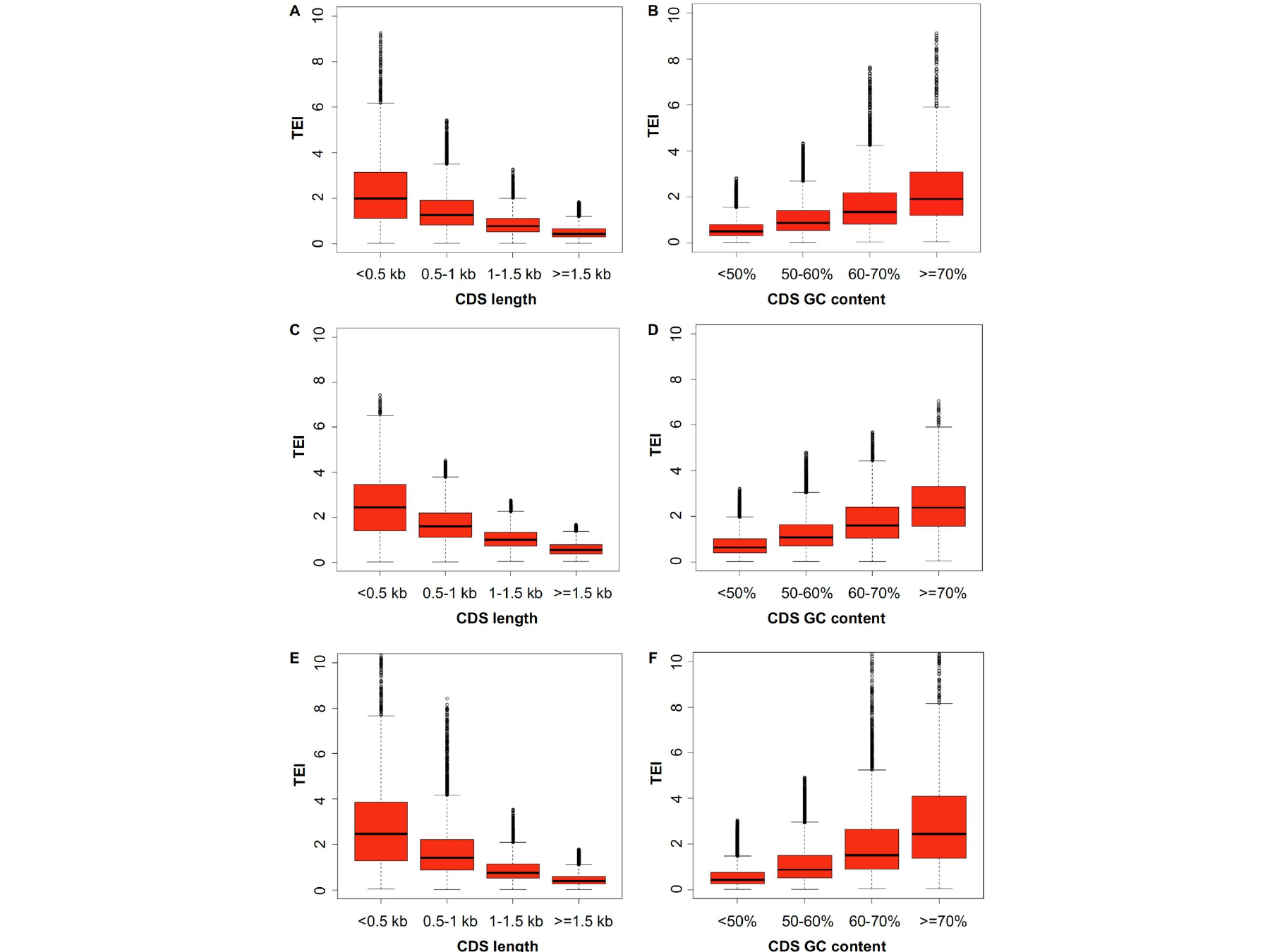

Supplement: Supplementary file 3 [file 203FigureS3.pptx]

## Slide 1
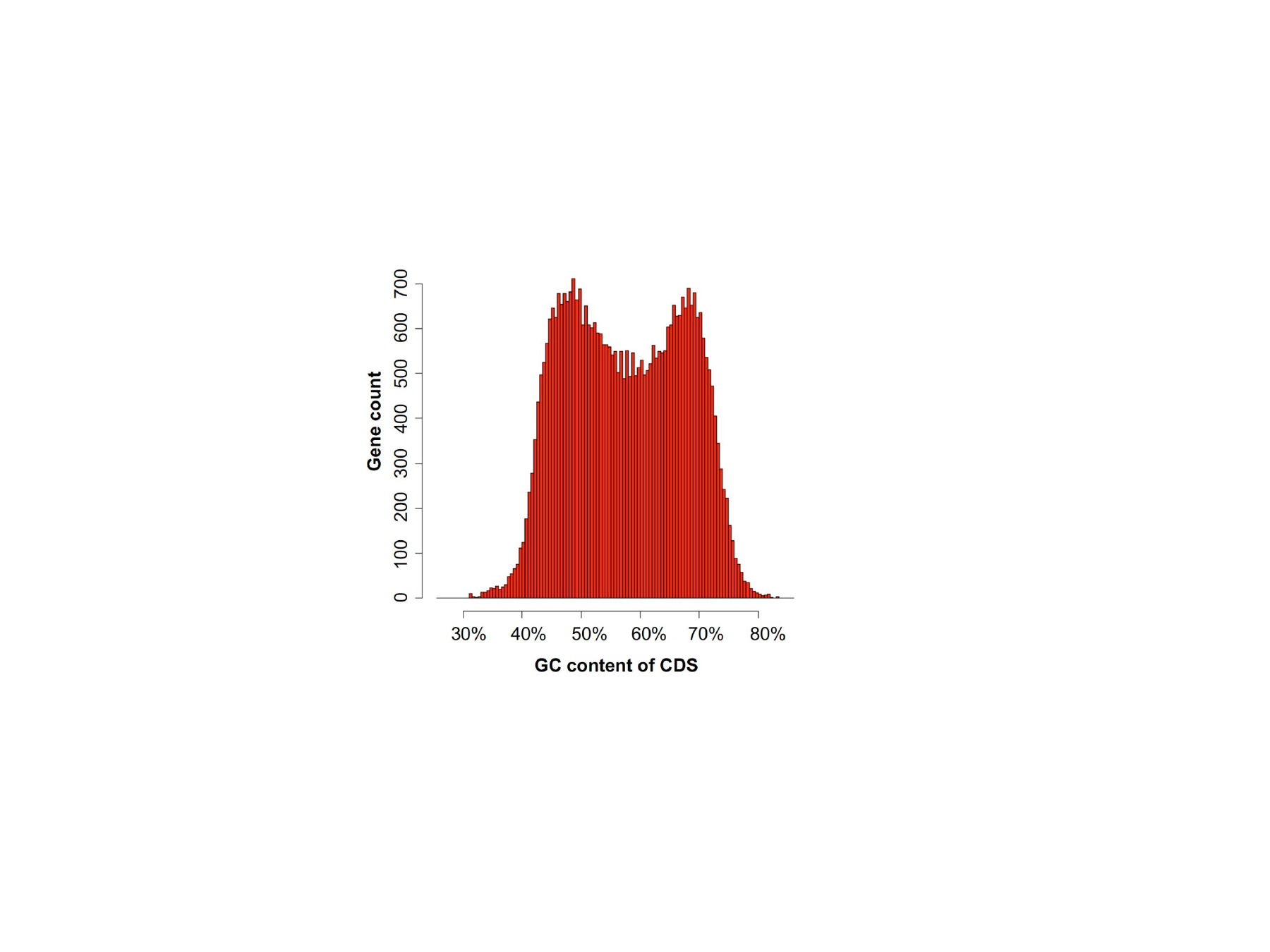

Supplement: Supplementary file 4 [file 203FigureS4.pptx]

## Slide 1
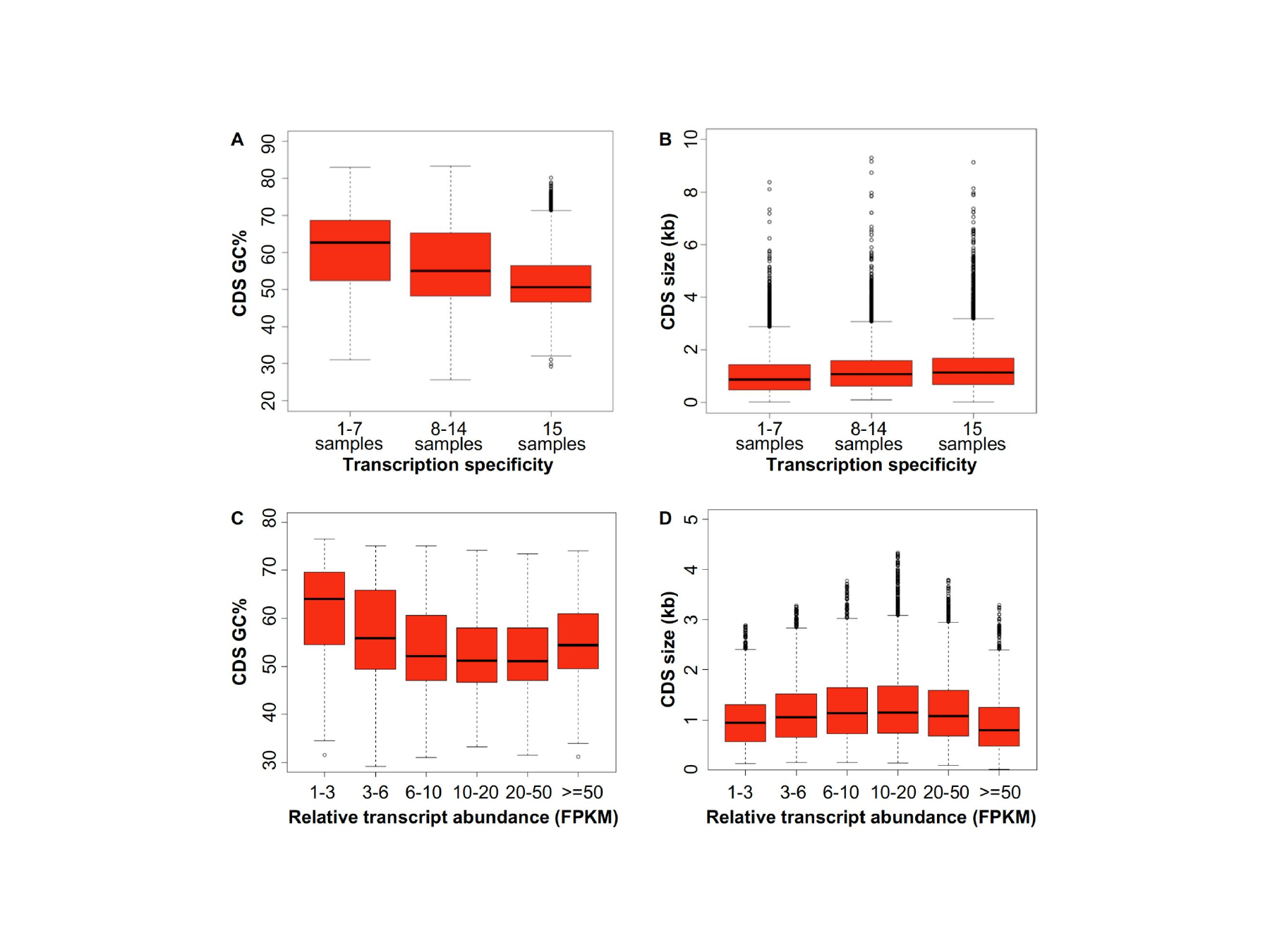

Supplement: Supplementary file 5 [file 203FigureS5.pptx]

## Slide 1
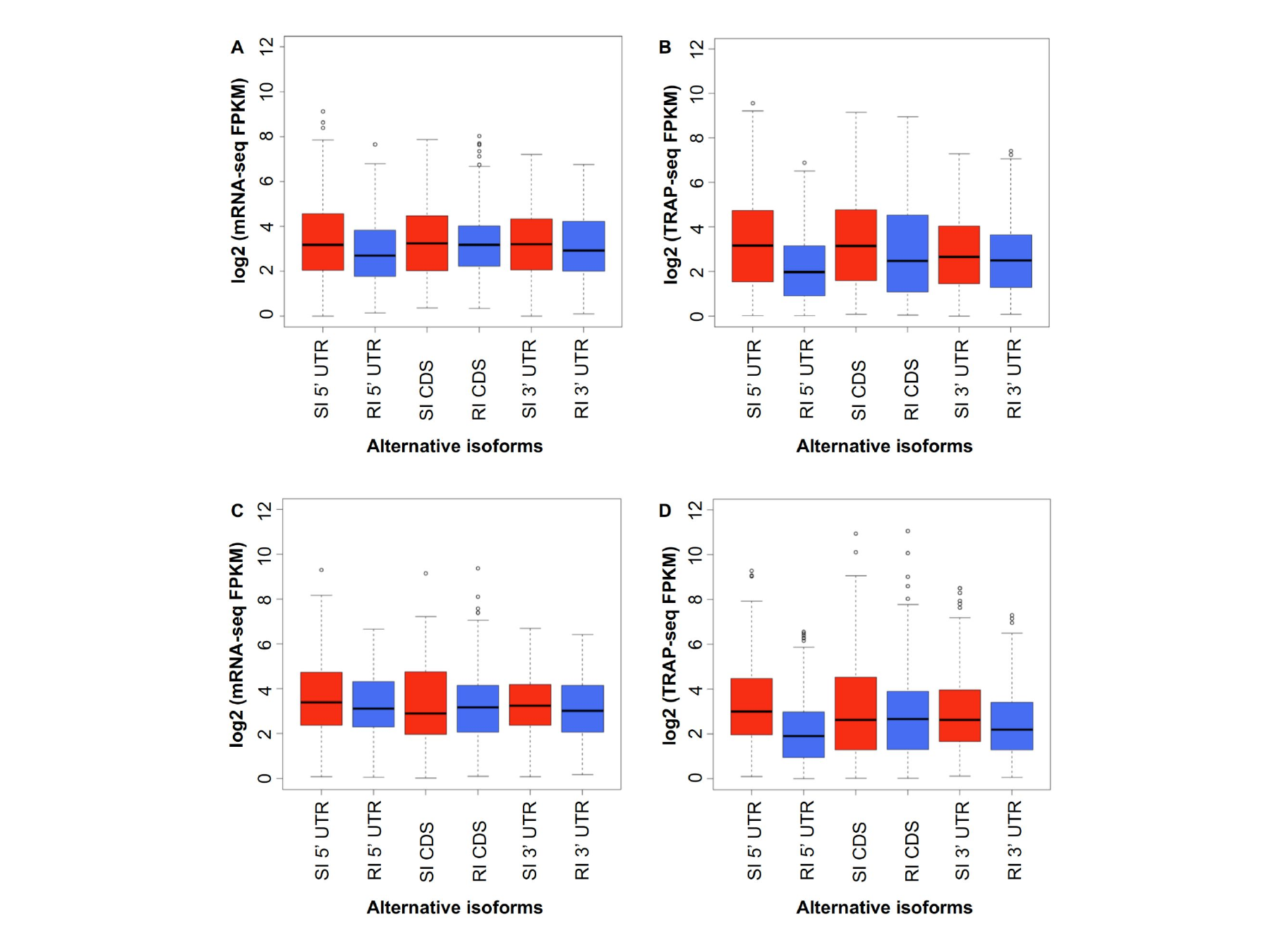

Supplement: Supplementary file 6 [file 203FigureS6.pptx]

## Slide 1
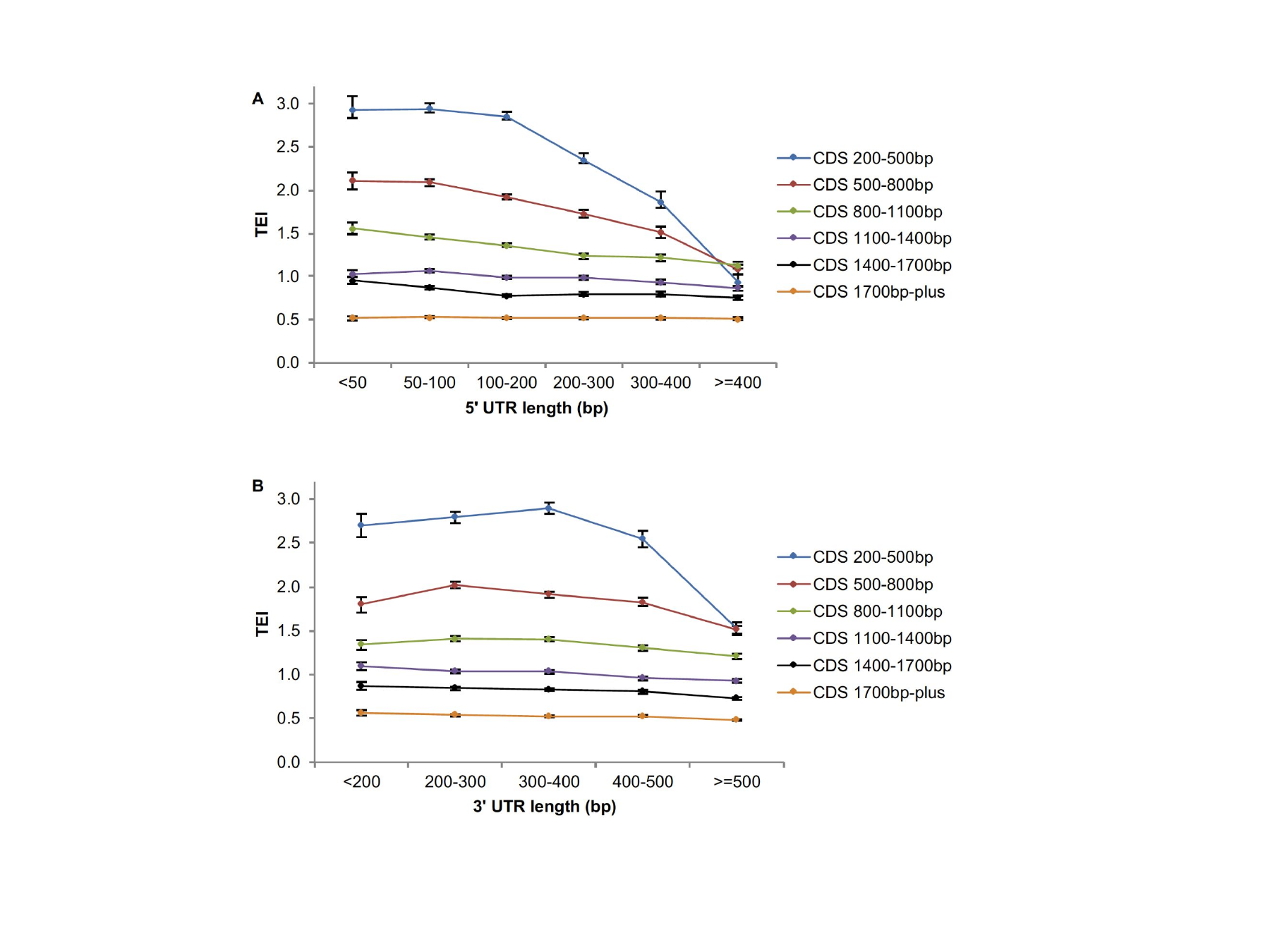

## Slide 2
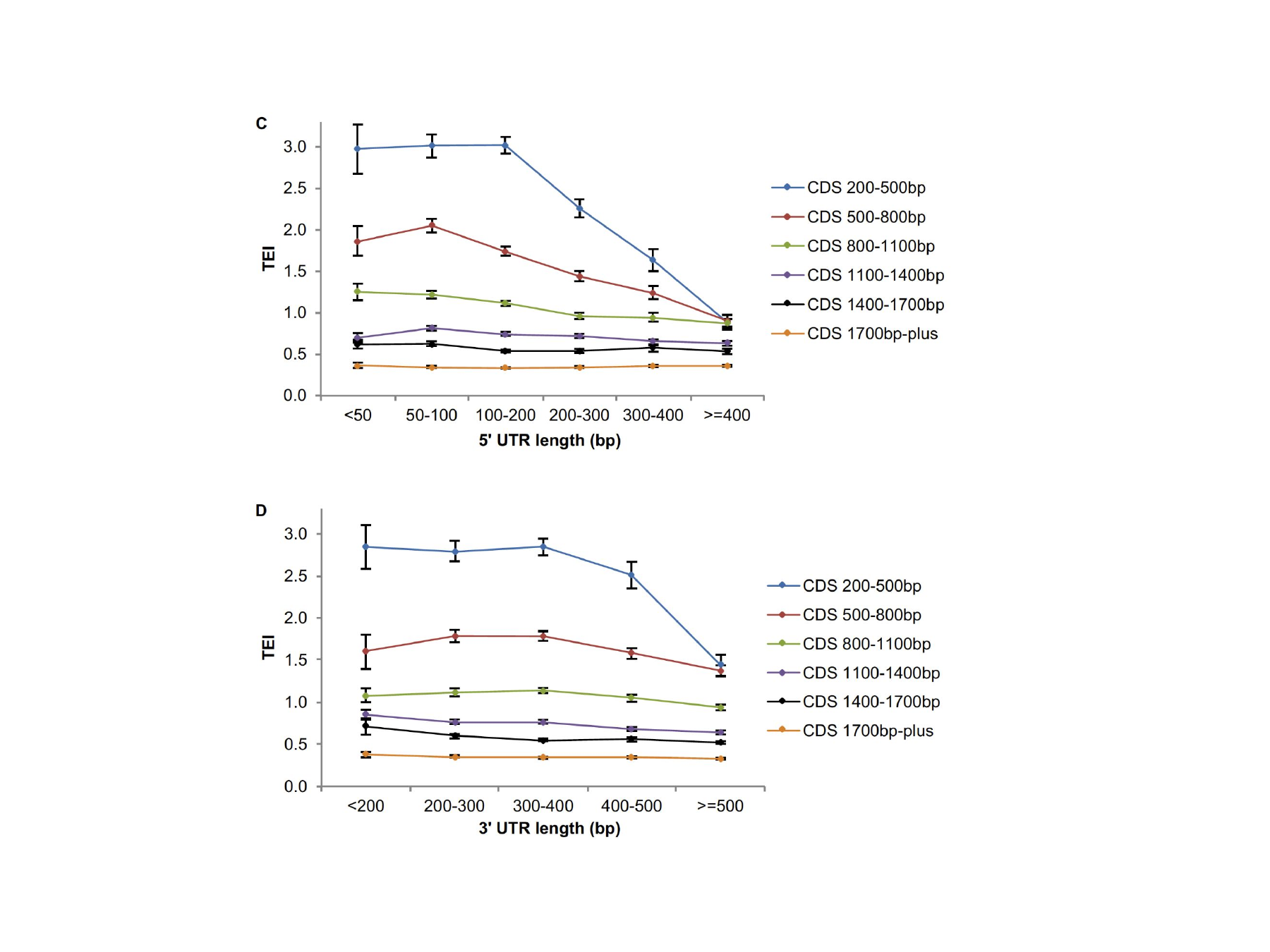

Supplement: Supplementary file 7 [file 203FigureS7.pptx]

## Slide 1
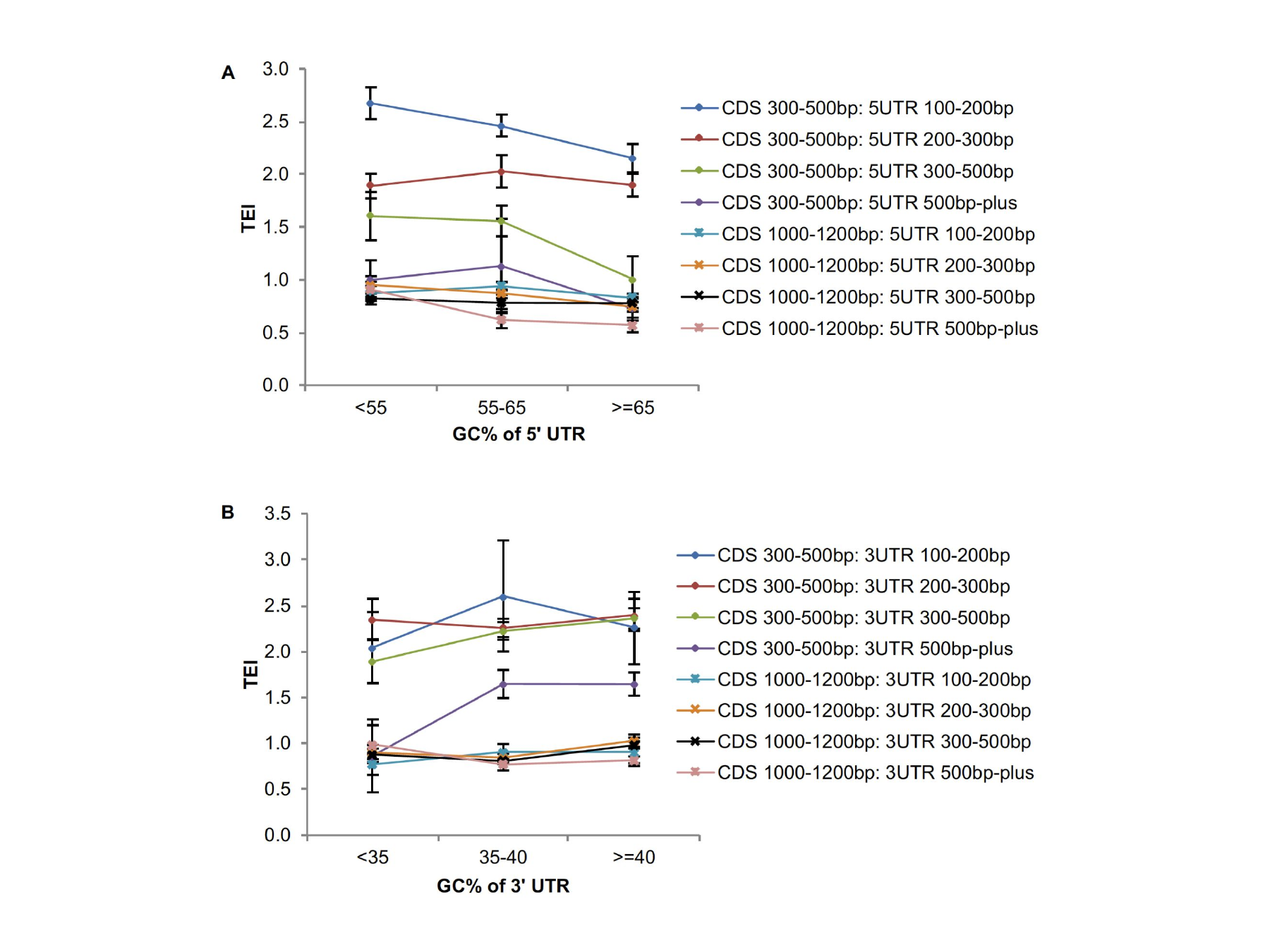

Supplement: Supplementary file 8 [file 203FigureS8.pptx]

## Slide 1
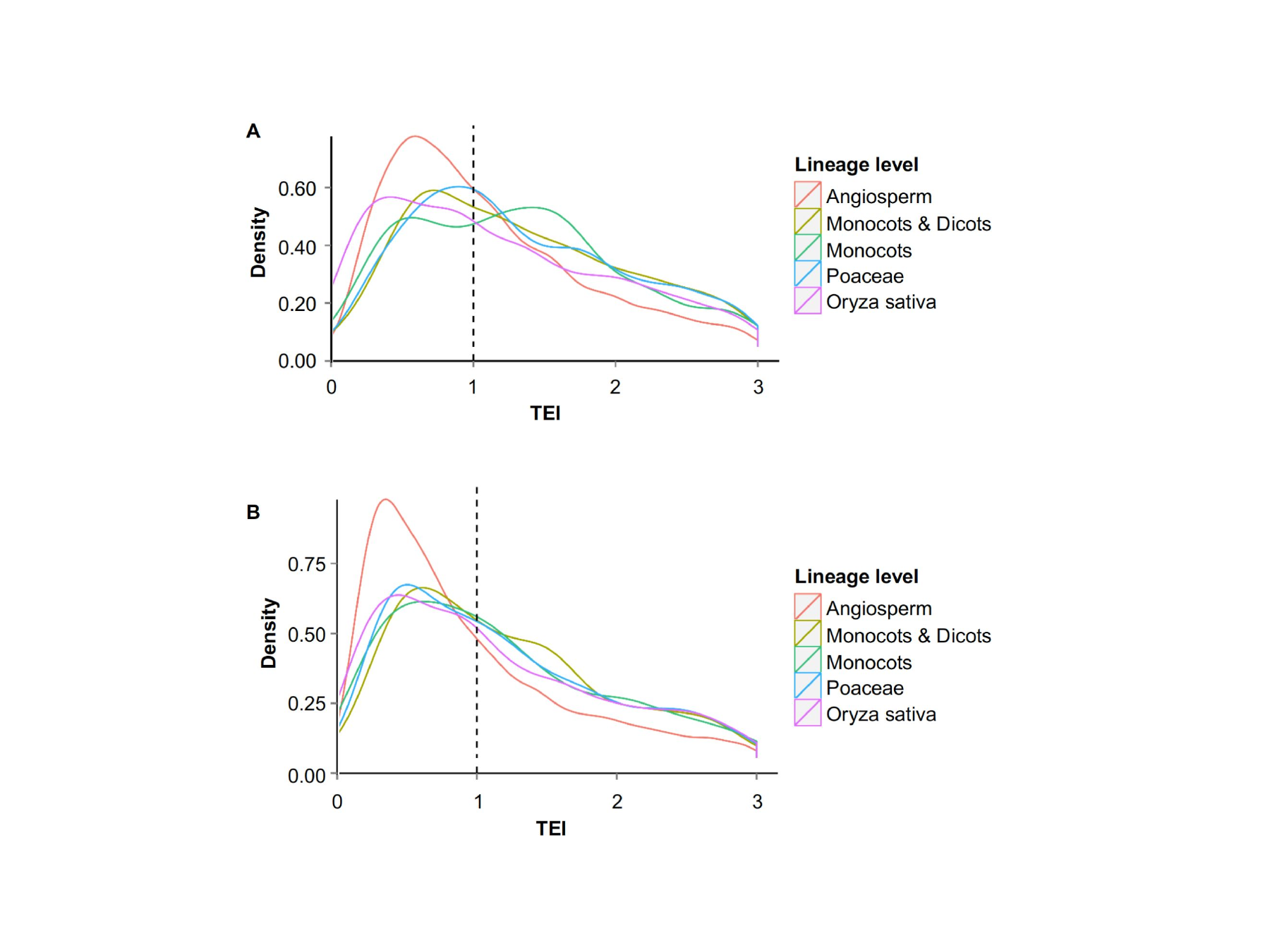

Supplement: Supplementary file 9 [file 203FigureS9.pptx]
