# Supplementary material for: Analysis of Ribosome-Associated mRNAs in Rice Reveals the Importance of Transcript Size and GC Content in Translation
Source: G3 (Bethesda). 2016 Nov 14;7(1):203–19. doi: 10.1534/g3.116.036020 (PMC5217110; doi:10.1534/g3.116.036020)
Supplement: Supplementary file 10 [file 203TableS1.docx]

**Table S1.** mRNA-sequencing and translating ribosome affinity purification followed by mRNA-sequencing (TRAP-seq) libraries generated in this study.

| SRA accession number | Sample description^a^ | Total number of raw reads | Percent of reads mapped to rice genome (%) |
| --- | --- | --- | --- |
| SRS1117497 | Shoot (transgenic RPL18) - mRNA-Seq | 45,690,146 | 97.1 |
| SRS1117525 | Shoot (transgenic RPL18) - TRAP-seq | 72,979,136 | 96.0 |
| SRS1117526 | Callus (transgenic RPL18) - mRNA-Seq | 95,654,544 | 97.0 |
| SRS1117524 | Callus (transgenic RPL18) - TRAP-seq | 62,983,896 | 95.3 |
| SRS1117523 | Panicles (transgenic RPL18) - mRNA-Seq | 175,465,992 | 97.9 |
| SRS1117527 | Panicles (transgenic RPL18) - TRAP-Seq | 89,889,104 | 97.4 |

^a^Shoot and callus libraries were sequenced in paired-end mode to 100 nt in length whereas the panicle libraries were sequenced in paired-end mode to 150 nt in length and trimmed to 100nt.
