# Supplementary material for: Analysis of Ribosome-Associated mRNAs in Rice Reveals the Importance of Transcript Size and GC Content in Translation
Source: G3 (Bethesda). 2016 Nov 14;7(1):203–19. doi: 10.1534/g3.116.036020 (PMC5217110; doi:10.1534/g3.116.036020)
Supplement: Supplementary file 13 [file 203TableS3.docx]

**Table S3.** mRNA-seq libraries used to determine expression breadth in this study.

| Sample ID | Description |
| --- | --- |
| SRS1117526 | *Oryza sativa*, Nipponbare, RPL18 callus |
| SRS1117497 | *Oryza sativa*, Nipponbare, RPL18 shoot |
| SRS1117523 | *Oryza* *sativa*, Nipponbare, RPL18 young panicle |
| SRX100741 | *Oryza sativa*, Nipponbare, 20 Day Leaves (OSN_AA) |
| SRX100743 | *Oryza sativa*, Nipponbare, Emerging Inflorescence (OSN_AB) |
| SRX100746 | *Oryza sativa*, Nipponbare, Anther (OSN_AD) |
| SRX100753 | *Oryza sativa*, Nipponbare, Embryo 25 Days After Pollination (OSN_AG) |
| SRX100754 | *Oryza sativa*, Nipponbare, Endosperm 25 Days After Pollination (OSN_AH) |
| SRX100755 | *Oryza sativa*, Nipponbare, Seed 10 Days After Pollination (OSN_AK) |
| SRX103305 | GSM823081: Seedling Biological Replicate 1; *Oryza sativa*; cDNA |
| SRX103307 | GSM823083: Callus Biological Replicate 1; *Oryza sativa*; cDNA |
| SRX330497 | *Oryza sativa*, Control-treated aleurone of seed |
| SRX332134 | *Oryza sativa*, Abscisic acid (ABA) treated aleurone of seed |
| SRX332135 | *Oryza sativa*, Gibberellic acid (GA) treated aleurone of seed |
| SRX332136 | *Oryza sativa*, Abscisic acid (ABA) and gibberellic acid (GA) treated aleurone of seed |
