# Supplementary material for: Analysis of Ribosome-Associated mRNAs in Rice Reveals the Importance of Transcript Size and GC Content in Translation
Source: G3 (Bethesda). 2016 Nov 14;7(1):203–19. doi: 10.1534/g3.116.036020 (PMC5217110; doi:10.1534/g3.116.036020)
Supplement: Supplementary file 14 [file 203TableS4.docx]

**Table S4.** Classification of genes based on their expression abundance

| Abundance group | Expression abundance | Shoot | No. of genes Callus | Panicle |
| --- | --- | --- | --- | --- |
| 1 | 1 ≥ FPKM < 3 | 2177 | 2506 | 2130 |
| 2 | 3 ≥ FPKM < 6 | 2556 | 2605 | 2386 |
| 3 | 6 ≥ FPKM < 10 | 2506 | 2507 | 2387 |
| 4 | 10 ≥ FPKM < 20 | 3355 | 3804 | 3860 |
| 5 | 20 ≥ FPKM < 50 | 2779 | 3624 | 3790 |
| 6 | FPKM ≥ 50 | 1657 | 1884 | 2251 |
