# Supplementary material for: Analysis of Ribosome-Associated mRNAs in Rice Reveals the Importance of Transcript Size and GC Content in Translation
Source: G3 (Bethesda). 2016 Nov 14;7(1):203–19. doi: 10.1534/g3.116.036020 (PMC5217110; doi:10.1534/g3.116.036020)
Supplement: Supplementary file 15 [file 203TableS5.docx]

Table S5. Genomic features of genes used to calculate the effect of length and GC content of different portions of transcripts on Translatome Enrichment Index (TEI). (.xlsx, 878 KB)

<http://www.g3journal.org/lookup/suppl/doi:10.1534/g3.115.020040/-/DC1/TableS5.xlsx>
