# Supplementary material for: Analysis of Ribosome-Associated mRNAs in Rice Reveals the Importance of Transcript Size and GC Content in Translation
Source: G3 (Bethesda). 2016 Nov 14;7(1):203–19. doi: 10.1534/g3.116.036020 (PMC5217110; doi:10.1534/g3.116.036020)
Supplement: Supplementary file 17 [file 203TableS6.docx]

**Table S6.** Correlation between CDS length and TEI, CDS GC content and TEI, CDS GC3 and TEI, CDS GC content and CDS GC3, and CDS length and GC content^a^.

|  | **CDS length vs. TEI** | | **CDS GC% vs. TEI** | | **CDS GC3**^b^ **vs. TEI** | | **CDS GC% vs. CDS GC3** | | **CDS length vs. CDS GC%** | |
| --- | --- | --- | --- | --- | --- | --- | --- | --- | --- | --- |
| CDS length ranges | correlation coefficient | p-value | correlation coefficient | p-value | correlation coefficient | p-value | correlation coefficient | p-value | correlation coefficient | p-value |
| 200-500 bp | -0.05939 | 0.004618 | 0.33611 | < 2.2e-16 | 0.40326 | < 2.2e-16 | 0.834 | < 2.2e-16 | 0.114448 | < 2.2e-16 |
| 500-800 bp | -0.18256 | < 2.2e-16 | 0.44575 | < 2.2e-16 | 0.43731 | < 2.2e-16 | 0.91390 | < 2.2e-16 | -0.072983 | 3.76E-10 |
| 800-1100 bp | -0.12621 | 3.06E-11 | 0.50471 | < 2.2e-16 | 0.46646 | < 2.2e-16 | 0.94254 | < 2.2e-16 | -0.024238 | 0.06971 |
| 1100-1400 bp | -0.07178 | 0.0004886 | 0.46870 | < 2.2e-16 | 0.42016 | < 2.2e-16 | 0.95148 | < 2.2e-16 | -0.014049 | 0.3317 |
| 1400-1700 bp | -0.14314 | 9.53E-10 | 0.53396 | < 2.2e-16 | 0.47651 | < 2.2e-16 | 0.95608 | < 2.2e-16 | -0.060190 | 0.0002669 |
| 1700 bp-plus | -0.32803 | < 2.2e-16 | 0.52010 | < 2.2e-16 | 0.47549 | < 2.2e-16 | 0.95671 | < 2.2e-16 | -0.275527 | < 2.2e-16 |

^a^Shading intensity increases with the increasing absolute value of correlation coefficient.

^b^GC content of the 3^rd^ codon positions of amino acids.
