# Supplementary material for: Analysis of Ribosome-Associated mRNAs in Rice Reveals the Importance of Transcript Size and GC Content in Translation
Source: G3 (Bethesda). 2016 Nov 14;7(1):203–19. doi: 10.1534/g3.116.036020 (PMC5217110; doi:10.1534/g3.116.036020)
Supplement: Supplementary file 18 [file 203TableS7.docx]

Table S7. Genomic features of genes used to calculate the correlation between CDS length and TEI, CDS GC content and TEI, CDS GC3 and TEI, CDS GC content and CDS GC3, and CDS length and GC content. (.xlsx, 950 KB)

<http://www.g3journal.org/lookup/suppl/doi:10.1534/g3.115.020040/-/DC1/TableS7.xlsx>
