# Supplementary material for: Analysis of Ribosome-Associated mRNAs in Rice Reveals the Importance of Transcript Size and GC Content in Translation
Source: G3 (Bethesda). 2016 Nov 14;7(1):203–19. doi: 10.1534/g3.116.036020 (PMC5217110; doi:10.1534/g3.116.036020)
Supplement: Supplementary file 20 [file 203TableS8.docx]

**Table S8.** The number of expressed genes, TEI, CDS length, and GC content of genes with known function compared with “expressed proteins/hypothetical proteins”

|  | Known genes | Expressed + hypothetical proteins |
| --- | --- | --- |
| Total genes | 22,896 | 16,153 |
| Transcribed | 16,879 (73.7%) | 5,610 (34.7%) |
| Translated | 16,151 (70.5%) | 5,496 (34.0%) |
| CDS length (median) | 1,179 | 615 |
| GC content (median) | 53.13 | 57.21 |
| TEI (median) | 0.88 | 1.14 |
