# Supplementary material for: Analysis of Ribosome-Associated mRNAs in Rice Reveals the Importance of Transcript Size and GC Content in Translation
Source: G3 (Bethesda). 2016 Nov 14;7(1):203–19. doi: 10.1534/g3.116.036020 (PMC5217110; doi:10.1534/g3.116.036020)
Supplement: Supplementary file 21 [file 203TableS9.docx]

**Table S9.** The translatome enrichment index (TEI), coding sequence (CDS) length, and GC content of 23 rice reference genes.

| Reference genes | Description | CDS size (bp) | CDS GC% | TEI in callus | TEI in panicle | TEI in shoot |
| --- | --- | --- | --- | --- | --- | --- |
| LOC_Os01g39260 | FtsH protease | 2148 | 47.49 | 0.466 | 0.267 | 0.462 |
| LOC_Os02g16040 | Ubiquitin | 447 | 50.11 | 2.829 | 2.295 | 1.283 |
| LOC_Os02g38920 | GAPDH | 1071 | 53.31 | 0.879 | 0.842 | 1.029 |
| LOC_Os02g46510 | AP-2 complex subunit | 1317 | 43.74 | 0.643 | 0.380 | 0.437 |
| LOC_Os03g21210 | endo-1,4-beta-glucanase | 1860 | 54.30 | 0.508 | 0.379 | 0.335 |
| LOC_Os03g25980 | Nucleotide tract-binding protein | 1395 | 50.11 | 1.189 | 0.848 | 0.667 |
| LOC_Os03g46770 | RNA-binding protein | 489 | 73.21 | 0.508 | 1.111 | 0.548 |
| LOC_Os03g50885 | Actin | 1134 | 54.76 | 1.016 | 1.071 | 0.831 |
| LOC_Os03g55270 | TIP41-like | 873 | 48.91 | 1.050 | 0.935 | 1.324 |
| LOC_Os05g36290 | Actin1 | 1131 | 47.21 | 1.150 | 0.578 | 1.044 |
| LOC_Os05g48960 | Splicing factor U2af | 915 | 54.75 | 0.785 | 0.825 | 0.522 |
| LOC_Os06g11170 | Nucleic acid binding protein | 732 | 46.04 | 1.174 | 1.109 | 0.607 |
| LOC_Os06g46770 | Polyubiquitin | 1602 | 58.61 | 1.862 | 2.352 | 1.956 |
| LOC_Os06g48970 | Protein kinase | 1098 | 44.90 | 0.851 | 0.679 | 0.567 |
| LOC_Os07g34589 | Translation factor SUI1 | 348 | 47.70 | 0.690 | 3.107 | 2.385 |
| LOC_Os07g38730 | Alpha-tubulin | 1353 | 52.99 | 0.881 | 0.657 | 0.663 |
| LOC_Os07g42300 | EF1d | 756 | 47.62 | 1.089 | 1.247 | 1.190 |
| LOC_Os07g43730 | EF1 | 195 | 47.18 | 2.357 | 3.669 | 2.110 |
| LOC_Os08g23180 | Arabinogalactan protein | 1248 | 68.59 | 1.133 | 1.249 | 0.903 |
| LOC_Os11g21990 | Eukaryotic initiation factor 5C | 1239 | 43.50 | 1.286 | 0.990 | 0.980 |
| LOC_Os11g26910 | SKP1-like protein 1A | 522 | 62.07 | 3.249 | 3.854 | 3.206 |
| LOC_Os11g43900 | Tumor protein homolog | 507 | 51.48 | 0.445 | 0.955 | 1.113 |
| LOC_Os12g32950 | Membrane protein | 201 | 47.26 | 3.573 | 5.333 | 1.994 |
| Median |  | **1071** | **50.11** | **1.05** | **0.99** | **0.98** |
