# Supplementary material for: Analysis of Ribosome-Associated mRNAs in Rice Reveals the Importance of Transcript Size and GC Content in Translation
Source: G3 (Bethesda). 2016 Nov 14;7(1):203–19. doi: 10.1534/g3.116.036020 (PMC5217110; doi:10.1534/g3.116.036020)
Supplement: Supplementary file 22 [file 203TableS10.docx]

**Table S10.** Translatome enrichment index (TEI) of spliced intron vs. retained intron (SI vs. RI) isoforms.

| Tissues | | | Callus | Panicle | Shoot |
| --- | --- | --- | --- | --- | --- |
| No. of pairs | | | 732 | 622 | 605 |
| TEI | Median | RI | 0.71 | 0.56 | 0.50 |
|  |  | SI | 0.99 | 0.83 | 0.84 |
|  |  | All non-TE genes | 1.05 | 0.87 | 0.86 |
|  | Mean | RI | 0.99 | 1.07 | 0.84 |
|  |  | SI | 1.30 | 1.64 | 1.26 |
|  |  | All non-TE genes | 1.36 | 1.43 | 1.24 |
