# Supplementary material for: Analysis of Ribosome-Associated mRNAs in Rice Reveals the Importance of Transcript Size and GC Content in Translation
Source: G3 (Bethesda). 2016 Nov 14;7(1):203–19. doi: 10.1534/g3.116.036020 (PMC5217110; doi:10.1534/g3.116.036020)
Supplement: Supplementary file 29 [file 203TableS14.docx]

Table S14. TEI, mRNA-seq level (FPKM), TRAP-seq level (FPKM), transcript size and GC content in of genes in all GO processes. (.xlsx, 23 KB)

<http://www.g3journal.org/lookup/suppl/doi:10.1534/g3.115.020040/-/DC1/TableS14.xlsx>
