# Supplementary material for: Analysis of Ribosome-Associated mRNAs in Rice Reveals the Importance of Transcript Size and GC Content in Translation
Source: G3 (Bethesda). 2016 Nov 14;7(1):203–19. doi: 10.1534/g3.116.036020 (PMC5217110; doi:10.1534/g3.116.036020)
Supplement: Supplementary file 35 [file 203TableS17.docx]

**Table S17.** Ten biological processes in which genes are associated with the highest Translatome Enrichment Index (TEI) in Arabidopsis seedlings^a^

| **GO processes with high TEI** | | | | | | |
| --- | --- | --- | --- | --- | --- | --- |
| GO term - process | TEI | mRNA-seq (FPKM) | TRAP-seq (FPKM) | CDS size (bp) | 5' UTR length | CDS GC content (%) |
| Photosynthesis | 1.14 | 894.9 | 1100.8 | 1065 | 108 | 46.7 |
| Translation | 1.10 | 163.9 | 221.5 | 1015 | 107 | 45.9 |
| Generation of precursor metabolites and energy | 1.09 | 426.7 | 593.3 | 1207 | 114 | 46.3 |
| Cellular homeostasis | 1.08 | 86.3 | 135.5 | 1273 | 141 | 45.4 |
| Biological process | 1.07 | 50.0 | 67.6 | 1165 | 146 | 45.0 |
| Response to abiotic stimulus | 1.05 | 190.2 | 224.2 | 1313 | 146 | 45.8 |
| Response to endogenous stimulus | 1.05 | 66.0 | 76.7 | 1376 | 172 | 45.6 |
| DNA metabolic process | 1.04 | 31.2 | 39.1 | 1321 | 128 | 44.6 |
| Response to biotic stimulus | 1.03 | 101.8 | 124.6 | 1469 | 143 | 45.8 |
| Response to stress | 1.03 | 110.7 | 130.0 | 1330 | 141 | 45.6 |
| **Mean** | **1.07** | **212.2** | **271.3** | **1253** | **135** | **45.7** |
|  |  |  |  |  |  |  |
| **GO processes with low TEI** | | | | | | |
| GO term - process | TEI | mRNA-seq (FPKM) | TRAP-seq (FPKM) | CDS size (bp) | 5' UTR length | CDS GC content (%) |
| Cell communication | 0.91 | 50.8 | 51.0 | 1526 | 167 | 45.2 |
| Pollination | 0.91 | 39.3 | 42.5 | 1646 | 157 | 44.9 |
| Response to extracellular stimulus | 0.91 | 48.8 | 48.7 | 1535 | 169 | 45.2 |
| Regulation of gene expression, epigenetic | 0.92 | 28.6 | 21.6 | 2163 | 149 | 44.5 |
| Carbohydrate metabolic process | 0.93 | 76.6 | 76.2 | 1621 | 149 | 45.0 |
| Cell cycle | 0.93 | 29.5 | 31.8 | 1722 | 169 | 44.1 |
| Catabolic process | 0.94 | 67.0 | 70.1 | 1586 | 143 | 45.1 |
| Lipid metabolic process | 0.94 | 50.0 | 51.4 | 1415 | 154 | 44.7 |
| Multicellular organismal development | 0.94 | 46.3 | 49.5 | 1647 | 172 | 45.1 |
| Cell differentiation | 0.97 | 44.3 | 49.8 | 1671 | 187 | 45.3 |
| **Mean** | **0.93** | **48.1** | **49.3** | **1653** | **162** | **44.9** |
| **Genome-wide Mean^b^** | **1.03** | **51.3** | **64.7** | **1267** | **146.0** | **45.1** |
| **Difference with high TEI processes** | **-0.14** | **-164.0** | **-222.1** | **400** | **27** | **-0.7** |
| **% Variation^c^** | **15.05** | **341.0** | **450.9** | **31.9** | **20.0** | **1.7** |

^a^Only processes with 40 or more genes are considered. The values are mean values in each process. ^b^Genome-wide mean is derived from non-TE genes with TEI and UTRs ≥ minimum (20 bp for 5' UTR and 110 bp for 3' UTR). ^c^%Variation equals difference divided by the lower values of the two means, then times 100. Shading intensity increases with the values in each column.
